# Selected heterozygosity at *cis*-regulatory sequences increases the expression homogeneity of a cell population in humans

**DOI:** 10.1186/s13059-016-1027-8

**Published:** 2016-07-28

**Authors:** Min Kyung Sung, Juneil Jang, Kang Seon Lee, Cheol-Min Ghim, Jung Kyoon Choi

**Affiliations:** 1 Department of Bio and Brain Engineering, KAIST, Daejeon, 34141 Republic of Korea; 2 School of Life Sciences, Ulsan National Institute of Science and Technology, Ulsan, 44919 Republic of Korea; 3 Department of Physics, Ulsan National Institute of Science and Technology, Ulsan, 44919 Republic of Korea; 4 Mathematical Bioscience Institute, The Ohio State University, Columbus, Ohio 43210 USA

## Abstract

**Background:**

Examples of heterozygote advantage in humans are scarce and limited to protein-coding sequences. Here, we attempt a genome-wide functional inference of advantageous heterozygosity at *cis*-regulatory regions.

**Results:**

The single-nucleotide polymorphisms bearing the signatures of balancing selection are enriched in active *cis*-regulatory regions of immune cells and epithelial cells, the latter of which provide barrier function and innate immunity. Examples associated with ancient *trans*-specific balancing selection are also discovered. Allelic imbalance in chromatin accessibility and divergence in transcription factor motif sequences indicate that these balanced polymorphisms cause distinct regulatory variation. However, a majority of these variants show no association with the expression level of the target gene. Instead, single-cell experimental data for gene expression and chromatin accessibility demonstrate that heterozygous sequences can lower cell-to-cell variability in proportion to selection strengths. This negative correlation is more pronounced for highly expressed genes and consistently observed when using different data and methods. Based on mathematical modeling, we hypothesize that extrinsic noise from fluctuations in transcription factor activity may be amplified in homozygotes, whereas it is buffered in heterozygotes. While high expression levels are coupled with intrinsic noise reduction, regulatory heterozygosity can contribute to the suppression of extrinsic noise.

**Conclusions:**

This mechanism may confer a selective advantage by increasing cell population homogeneity and thereby enhancing the collective action of the cells, especially of those involved in the defense systems in humans.

**Electronic supplementary material:**

The online version of this article (doi:10.1186/s13059-016-1027-8) contains supplementary material, which is available to authorized users.

## Background

In diploid genomes, there are loci for which heterozygotes have higher fitness than either homozygote. Heterozygote advantage can maintain advantageous diversity against random genetic drift and can be considered a classic type of balancing selection. Well-known examples include the selective advantage of heterozygous mutants for the hemoglobin gene [1, 2] and the CFTR gene [3]. Additionally, heterozygosity at the MHC (major histocompatibility complex) loci may enhance resistance to a wider range of infections and increase survival rates [4, 5]. Recent population-based association studies have uncovered a heterozygote advantage associated with the Klotho gene [6, 7]. A recent study [8] proposed a possible evolutionary link between monoallelic expression and heterozygote advantage. Monoallelic expression leads to the formation of distinct cell subpopulations, depending on which protein-coding allele is active and which allele is downregulated [9]. The increased cell-to-cell heterogeneity is thought to be the advantage subjecting these alleles to balancing selection [8].

While all of these examples are limited to protein-coding regions, regulatory variations can also be targets of balancing selection. Almost all *trans*-specific balanced polymorphisms in humans and chimpanzees are in non-coding regions [10] and there are substantial examples of balancing selection on *cis*-regulatory regions [11, 12]. According to a theoretical model, regulatory mutations with a large effect can be adaptive in heterozygotes because their effect is moderated, thus preventing the overshooting of the optimal level of gene expression [13].

Balancing selection leaves genetic signatures that appear as an excess of polymorphisms at intermediate frequencies compared with the expectations under neutrality. This pattern can be detected using Tajima’s *D* test [14] or the Hudson–Kreitman–Aguadé (HKA) test [15, 16]. A Tajima’s *D* > 0 or an HKA k > 1 indicates balancing selection. Balancing selection maintains the same alleles in different populations and thus lowers population differentiation, which can be detected by Wright’s Fst test [17]. Within a population, different alleles at the same locus acquire their own set of neutral mutations and thus cause differentiation between haplogroups [18].

## Results and discussion

**Fig. 1:**
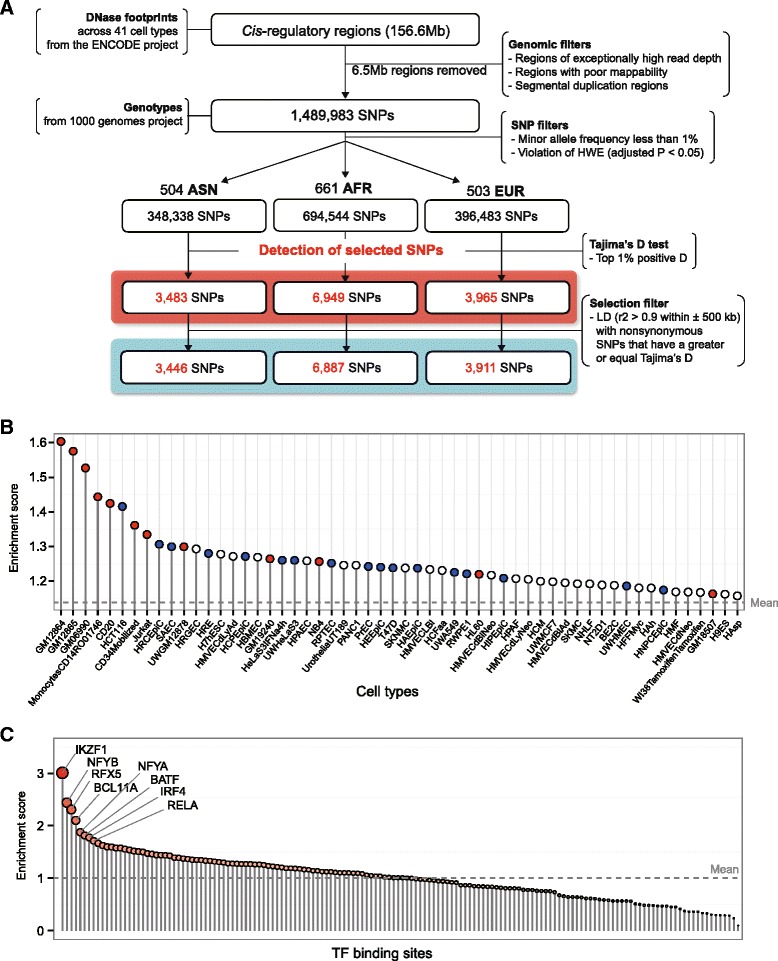
Identification of balanced SNPs and their enrichment in cell type-specific DNase hypersensitive sites (DHSs) and transcription factor (TF) binding sites. **a** Our data processing workflow for the identification of *cis*-regulatory SNPs that have potentially been under balancing selection. *AFR* African, *ASN* Asian, *EUR* European, *HWE* Hardy–Weinberg equilibrium , *LD* linkage disequilibrium. **b** Enrichment of the balanced SNPs in the DHSs of 125 cell types. Among them, only the cell types in the top half are displayed. An enrichment score reflects the degree to which a set of SNPs is overrepresented in a specific cell type. Immune cells and epithelial cells are highlighted in *red* and *blue*, respectively. The *dashed line* indicates the mean level of enrichment across the 125 cell types. **c** Enrichment of the balanced SNPs in the binding regions of 161 different TFs. The *dot size* is proportional to the degree of enrichment. The *dashed line* corresponds to the mean value

We searched DNase footprints for balancing selection signatures in three different populations with various filters that discarded artifactual single-nucleotide polymorphisms (SNPs) (Fig. 1a). The DNase footprint SNPs with the largest (top 1 %) positive Tajima’s *D* values in each population were chosen (Additional file 1: Figure S1). The selected regions had significantly high HKA k values (95.4 % having k > 1) and low Fst values (Additional file 1: Figure S2). The increased diversity was associated with high divergence between haplogroups compared with within haplogroups [18] (Additional file 1: Figure S3). Only a minor fraction (Asian, 1.12 %; African, 0.85 %; European, 1.36 %) of the selected regulatory SNPs were in linkage disequilibrium (LD) with non-synonymous SNPs. We discarded the cases in which the non-synonymous SNPs had a higher Tajima’s *D* than the matched regulatory SNPs (Additional file 1: Figure S4).

The balanced SNPs were enriched in the *cis*-regulatory regions that are active in immune cells, such as B cells, T cells, monocytes, and NK cells (red dots in Fig. 1b), or in epithelial cells, such as intestinal and airway epithelial cells (blue dots in Fig. 1b) (Additional file 1: Figure S5; Additional file 2). Binding sites for transcription factors (TFs) involved in immune function were also enriched (Fig. 1c; Additional file 1: Figure S6). We mapped the target genes of the selected SNPs (see “Methods”). Genes involved in epidermal development, keratinization, or antigen processing were enriched (Additional file 3). This enrichment is partly attributed to the clusters of the small proline-rich protein (SPRR) genes, late cornified envelope (LCE) genes, and MHC class II genes (Additional file 1: Figure S7). As structural components of insoluble cell envelopes, the SPRR and LCE proteins function to provide barriers in different epithelial cells, including epidermal keratinocytes, against various environmental challenges [19, 20].

**Fig. 2:**
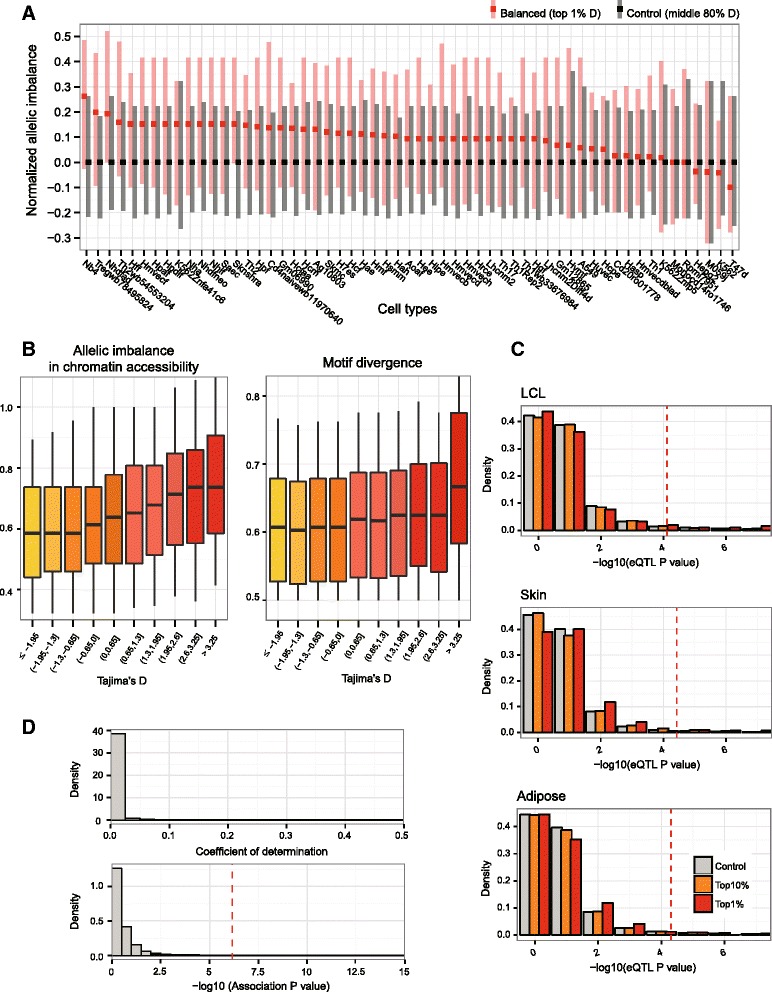
Low expression variation notwithstanding high regulatory sequence divergence at balanced SNPs. **a** The distribution of allelic imbalance calculated as the ratio of two alleles from the reads of chromatin accessibility for the balanced (*red*) or control (*black*) SNPs at heterozygous loci. In each cell type, the imbalance measures were normalized by subtracting the median of the control SNPs. *Colored squares* indicate the median point of the distribution and the *box* extends from the 30th to 70th percentile. **b** Allelic imbalance in chromatin accessibility (*left*) and allelic divergence in TF motif sequences (*right*) as a function of the Tajima’s *D* of the footprint SNPs. A higher motif divergence reflects more unique motifs for each allele. **c** Significance of expression quantitative trait loci (*eQTL*) mapping in lymphoblastoid (*LCL*), skin, and adipose cells according to the selection strengths at the regulatory SNPs. The *red dotted lines* denote an adjusted *P* value of 0.05. **d** The distribution of the coefficients of determination (*R* ^*2*^; *top*) and the *P* value (*bottom*) from a linear regression between the genotype of the balanced SNPs and the expression level of their target genes in whole-genome sequenced samples. The *red vertical line* indicates the adjusted *P* value of 0.05

If different alleles at the same locus were indeed maintained under selection, the functional differentiation of the alleles should appear. We first tested whether the different alleles resulted in differential chromatin accessibility. For this, we looked for a bias in the number of sequencing tags harboring each allele at the heterozygous DNase footprints. This tendency of allelic imbalance was specifically observed at the selected sites in many different cell types (Fig. 2a). There was a positive correlation between the degrees of selection and the allelic imbalance (Fig. 2b, left). In addition, the allelic divergence in motif sequences suggested that different sets of TFs bind to each allele at the selected sites (Fig. 2b, right). More empirical evidence for the allelic binding differences was provided by enrichment of the balanced SNPs for binding quantitative trait loci (QTL) of five TFs [21] (Additional file 4).

However, the regulatory sequence divergence was not reflected in variations in gene expression. Expression QTL (eQTL) mapping failed to reveal target gene expression significantly associated with the selected SNPs (Fig. 2c). For example, in lymphoblastoid cells, only 3.43 % of the balanced (Tajima’s *D* top 1 %) SNPs had an adjusted *P* value < 0.05. In addition to eQTL mapping, we examined the direct association between the selected SNPs and their target genes by leveraging the base-pair resolution genotypes of 358 individuals (see “Methods”). Again, the association was not strong, with only 2.25 % of the selected SNPs having an *R*^*2*^ > 0.2 or an adjusted *P* value < 0.05 (Fig. 2d). The significant SNPs were eliminated from the following analyses. These findings suggest that the allelic divergence did result in differential TF binding but not in differential gene expression.

**Fig. 3:**
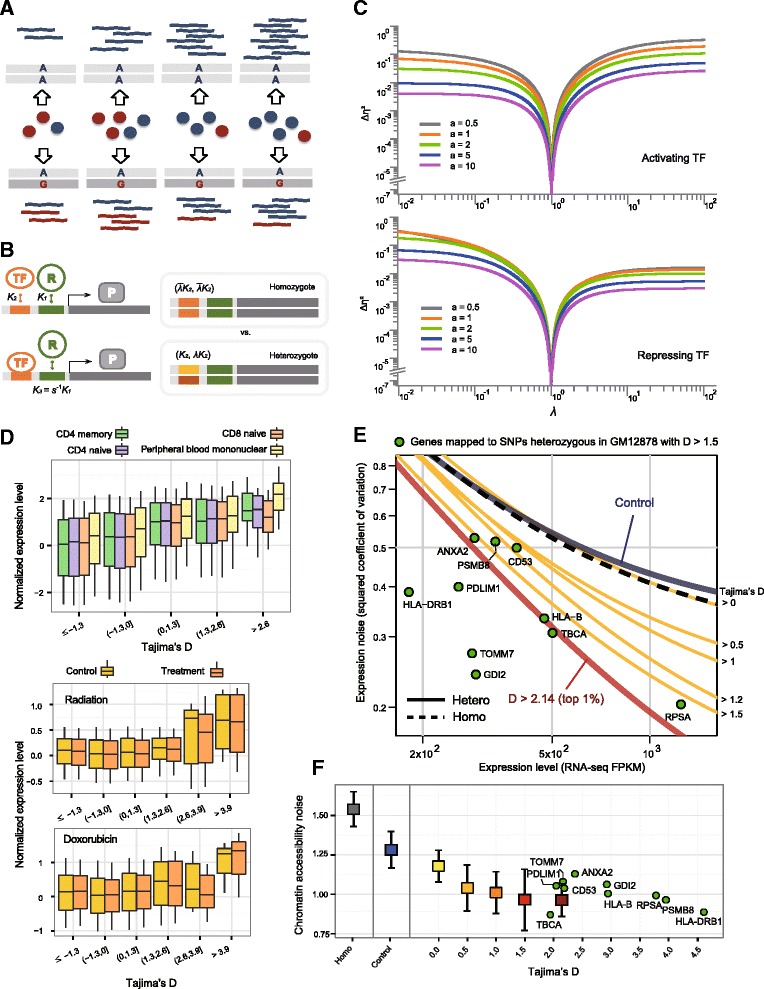
Correlation of selection for heterozygotes with cell-to-cell variation in gene expression and chromatin accessibility. **a** Illustration of how heterozygosity can buffer stochastic noise caused by the fluctuations of binding regulators. *Red* and *blue spheres* indicate TFs binding to the G and A alleles, respectively. Variation in the blue RNAs directly reflects variation in the blue TFs across the four homozygous cells (*top*), whereas variation in the red RNAs compensates for variation in the blue RNAs among the heterozygotes (*bottom*). It is assumed that the sources of extrinsic noise affecting the two alleles are uncoupled. **b** Our mathematical model detailed in the “Methods”. *K*_1_ is the dissociation constant between the promoter and RNA polymerase (*R*). *K*_2_ is the dissociation constant between the *cis*-regulatory region and TF. For repressing TFs, *K*_3_ is used in place of *K*_1_. The noise levels in protein expression are compared between the heterozygote with (*K*_2_, *λK*_2_) and the homozygote with $\left(\overline{\lambda}{K}_2,\overline{\lambda}{K}_2\right)$, given the same level of average gene expression. **c** The differences in the squared coefficient of variation (CV^2^) between the heterozygote and homozygote were obtained as a function of the allele discrepancy parameter *λ* for the varying TF concentration parameter *a*. As *λ* deviates from one, the two alleles of the heterozygote are more differentially regulated. Whether the TF is an activator (*top*) or repressor (*bottom*), the noise differences ∆*η* ^2^ were constantly greater than zero, indicating that the homozygote introduces higher levels of transcriptional noise than the heterozygote. **d** Gene expression level as a function of the Tajima’s *D* of the associated regulatory SNP. RNA sequencing data in four white blood cells from the Roadmap Epigenomics project (*top*) and DNA microarray data in GM12878 before and after particular treatments (*bottom*) were normalized (see “Methods”) before plotting. **e** Progressive reduction of the expression noise (CV^2^) in proportion to the Tajima’s *D* of the associated regulatory SNP. The curves show a fit to RNA sequencing data for 62 single cells with the *solid* and *dashed lines* representing heterozygous and homozygous loci in the GM12878 cells, respectively. The heterozygous curves are divided based on Tajima’s D. The *green dots* indicate representative genes that have a detectable RNA sequencing measure and are mapped to footprint SNPs heterozygous in GM12878 with *D* > 1.5. **f** Observed cell-to-cell variability in the chromatin accessibility of the *cis*-regulatory regions categorized in the same manner as in **e**. *Error bars* represent one standard deviation of the variability obtained through bootstrapping (see “Methods”)

If the level of gene expression is not the resulting phenotype, frequency-dependent selection, as well as heterozygote advantage due to moderation of expression levels [13], can be ruled out. Natural selection acting on stochastic noise or cell-to-cell variation in gene expression has been demonstrated in yeast [22, 23]. It was suggested that some TF binding QTLs that were not eQTLs might affect phenotypes only by affecting transcriptional variability between cells [21]. There are two types of cell-to-cell variability or noise. Intrinsic noise is the variability that typically originates from fluctuations that are inherent to biochemical processes such as promoter binding. Extrinsic noise mostly arises from cell-to-cell differences in shared cellular factors. A major source of extrinsic gene expression noise is stochastic fluctuations in the level of upstream transcription regulators [24]. We hypothesized that these fluctuations might be amplified in homozygotes, whereas divergent heterozygous sequences might reduce this effect because fluctuations in TFs binding to one allele and fluctuations in TFs specific to the other allele are not likely to be in the same phase, especially when these two sets of TFs are independent of each other (Fig. 3a).

In our mathematical model, we quantified the discrepancy in regulator binding between the two alleles and examined the difference in extrinsic noise between homozygotes and heterozygotes (Fig. 3b). When there was a certain level of allele discrepancy, the noise levels were consistently higher in homozygotes than heterozygotes for a varying TF fluctuation parameter (Fig. 3c). However, as can be inferred from our model and equations (“Methods”), this noise difference may not be recognizable in effect when intrinsic noise predominates, which is the case with genes expressed at a low level [25, 26]. Therefore, if the footprints of balancing selection we identified are related to extrinsic noise, the balanced SNPs should be linked to highly expressed genes. This turned out to be the case when we examined gene expression levels as a function of Tajima’s *D* in various immune cells (Fig. 3d).

**Fig. 4:**
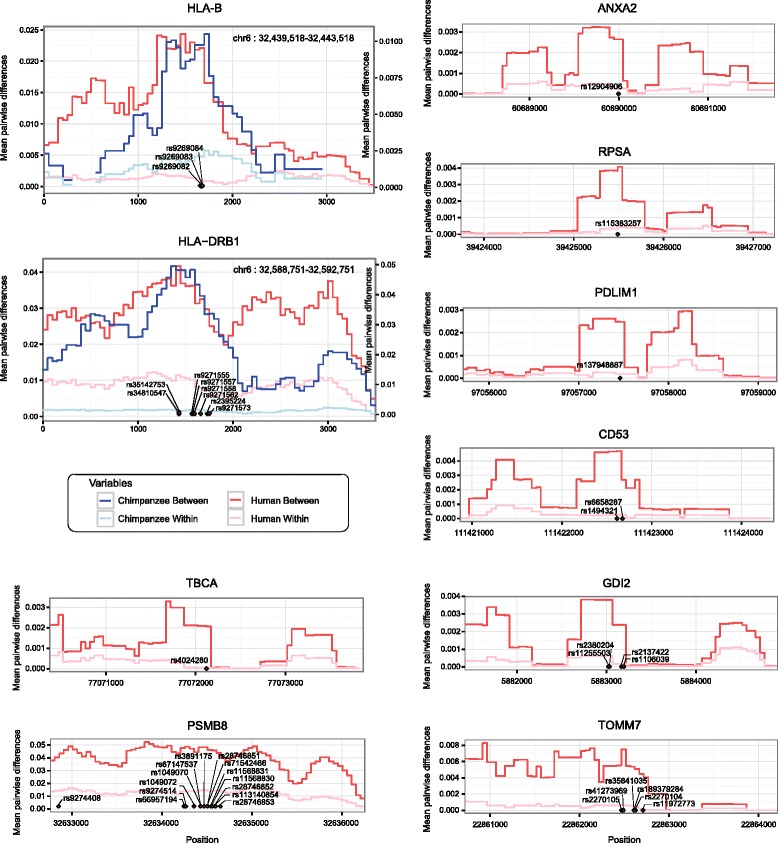
Selection signature at regulatory SNPs of representative genes. The mean pairwise differences between allelic classes (*dark red*) and within an allelic class (*light red*) for the regulatory sequences with Tajima’s *D* > 1.5 and whose patterns of noise in chromatin variability and associated gene expression are shown in Fig. 3e, f (*green dots*). The pairwise differences were calculated in 500-bp sliding windows. The pairwise differences between haplogroups compared with those within haplogroups are plotted. The between-group (*dark blue*) and within-group (*light blue*) pairwise differences near the HLA-B and HLA-DRB1 SNPs in chimpanzees are shown on the *right*-*hand y*-*axis*

To obtain empirical evidence, we used single-cell RNA and chromatin sequencing data in GM12878 lymphoblastoid cells (see “Methods”). The genes with stronger selection signatures at their *cis*-regulatory loci were expressed with lower noise (CV^2^) than the genes with homozygosity or weakly selected heterozygosity (Fig. 3e for Tajima’s *D* and Additional file 1: Figure S8a for HKA k). This negative correlation was quantified using the noise strength (R = −0.185 for Tajima’s *D* and R = −0.313 for HKA k; Additional file 1: Figure S9). Using a different enhancer–promoter mapping dataset recapitulated the same pattern (Additional file 1: Figure S10). If the observed patterns are caused by TF fluctuations, they should be reflected in the cell-to-cell variation in chromatin accessibility. Indeed, GM12878 single-cell chromatin accessibility data showed a negative correlation between the chromatin noise and selection strengths (Fig. 3f for Tajima’s *D* and Additional file 1: Figure S8b for HKA k). Green dots in Fig. 3e, f indicate representative genes with Tajima’s *D* > 1.5. The divergences between haplogroups harboring these SNPs are shown in Fig. 4. Of these, the HLA-B SNPs and HLA-DRB1 SNPs are segregating commonly in chimpanzees and in the three human subpopulations analyzed, offering an example of ancient, long-lived balancing selection [10].

Finally, we tested whether the selective advantage of regulatory heterozygosity lies in transcriptional flexibility. Heterozygous *cis*-regulatory sequences may be capable of responding to more diverse environmental cues because of their increased repertoire of binding regulators. To test this possibility, we employed data for gene expression or chromatin changes in response to various treatments in GM12878 cells. We failed to observe any differential capability of the selected heterozygous *cis*-regulatory sequences in responding to external cues (Additional file 1: Figure S11).

It is difficult to directly demonstrate that the proposed mechanism of noise suppression has conferred a selective advantage during the course of human evolution. Nonetheless, the progressive reduction of both chromatin and expression noise in proportion to the strength of the selection signature strongly suggests that the noise levels have been subject to selection. Robust gene expression in immune cells will increase the number of effective defensive cells in an organism. Epithelial cells, including intestinal or respiratory epithelial cells and keratinocytes, are positioned at the interface between the outside environment and the body interior, functioning as both a physical barrier and as immune sentinels. These cells express MHC class II molecules for communication with the immune system [27]. Hence, noisy gene expression in epithelial cells will weaken this barrier function.

One cannot rule out the possibility that heterozygotes gain a selective advantage through moderation or diversification of transcriptional response. Some of the balanced SNPs that we identified must be subject to these types of selection. First, although expression association was not observed in cell culture conditions, it is possible that variation in regulatory sequences may be manifested only under specific conditions. Second, while our tests for transcriptional flexibility were limited to particular types of stimuli, heterozygotes at different loci may readily respond to different environmental cues. In these two cases, the advantage of each regulatory SNP will be evident only in response to its associated specific environmental cue. In contrast to these conditionally controlled loci, constitutively active regulatory regions may be subject to balancing selection for noise minimization. Essential genes may be highly expressed because they are subject to selection to reduce noise whereas active gene expression can reduce intrinsic noise [25, 26]. Regulatory heterozygosity may confer a selective advantage especially on these genes by buffering extrinsic noise.

## Conclusions

Examples of heterozygote advantage in humans were confined to protein-coding sequences. By levearing a wealth of recent epigenomic data, we were able to perform a genome-wide functional inference of advantageous heterozygosity at cis-regulatory regions. Single-cell experimental data and mathematicl modeling demonstrated that heterozygous sequences can lower cell-to-cell variability in proportion to selection strengths and led to the hypothesis that extrinsic noise from fluctuations in TF activity may be amplified in homozygotes, whereas it is buffered in heterozygotes. Noise reduction by this mechanism may confer a selective advantage by increasing cell population homogeneity and thereby enhancing the collective action of the cells, especially of those involved in the defense systems.

## Methods

### Data processing for the identification of balanced SNPs

To detect the signature of balancing selection, we used the genotype information from the East Asian (ASN, n = 504), African (AFR, n = 661), and European (EUR, n = 503) panels from the 1000 Genomes Project [28] with a focus on SNPs in *cis*-regulatory or TF binding regions. Our workflow of selection signature detection is shown in Fig. 1a. For the accurate identification of *cis*-regulatory regions, we used DNase footprint data across 41 cell types from the ENCODE Project [29]. Nucleotide resolution analysis of the DNase cleavage patterns enabled us to discover the footprints of binding TFs ranging in size from 6 to 40 bp. The mean size of the footprint was 15 bp and the median was 9 bp [30].

We sought to eliminate the potential effects of non-allelic nucleotide variations among duplicates or repetitive sequences on nucleotide diversity patterns, based on which balanced polymorphisms can be detected (described in the next section). First, highly similar sequences collapsed in the reference genome assemblies (GRCh37/hg19) will result in an unusually high mapping depth. Therefore, to exclude regions with an exceptionally high depth of aligned short reads and poor mappability, we used the depth information inferred from the alignment of short-read sequences from the 1000 Genomes Project [31]. Specifically, the SNPs whose 50-kb flanking regions overlapped the regions of the top 0.1 % of the mapping depth were filtered out. We also applied a mappability filter to discard SNPs for which any 50-mer overlapping the site could be mapped to more than one location allowing up to two mismatches. The CRG 50-bp mappability score [32] was available from the UCSC Genome Browser. We also excluded putative genomic duplicated regions (≥1 kb and ≥90 % identity) [33, 34], which were not collapsed in the reference genome because of relatively lower sequence similarity. These correctly annotated duplicates will not cause an unusually high mapping depth but can cause false allelic variant calls due to the incorrect mapping of some reads from paralogues.

In addition, we discarded SNPs with minor allele frequencies (MAFs) less than 1 %, as well as SNPs that violated the Hardy–Weinberg equilibrium (HWE) [35]. A Bonferroni adjusted *P* value of 0.05 was used as the threshold of the HWE test. It should be noted that, under strong heterozygote advantage, we should observe an excess of heterozygous individuals at sites in the vicinity of the site under balancing selection. In other words, a balancing selection signal could be lost due to such filtering because deviations from the HWE are expected under heterozygote advantage. However, selection required to cause HWE violation as distinctly as at the adjusted *P* value < 0.05 should be extremely strong, and such strong selection would be detected using almost any methods. Moreover, while the methods for detecting balancing selection capture the genetic signatures of selection in the past, deviation from the HWE reflects selection acting in the present-day population. Thanks to medical advances, many selective disadvantages are not so fatal in the contemporary population as they were in the past. Therefore, HWE filtering will miss only a minor fraction of truly balanced SNPs. Well-established examples of balancing selection in the human genome, such as the selection in the MHC region, are not lost because of HWE filtering [36]. In essence, our study observes genome-wide patterns; thus, losing a small portion of balancing signatures is not critical. On the other hand, the major concern with sequencing data is mapping error; thus, HWE filtering is necessary to reduce the confounding effects of regions with these bioinformatic artifacts and the problems caused by many unidentified paralogues in the human genome.

### Statistical analysis for detection of balancing signatures

For screening the departure signals from the expected patterns of neutral variation, we calculated the Tajima’s *D* statistic [14] for the 5-kb regions centered on focal SNPs residing within DNase footprint regions. Tajima’s *D* is computed based on the difference between the mean number of pairwise differences and the number of segregating sites. These two diversity measures are scaled such that they are expected to be the same under neutrality. Excess polymorphisms at intermediate frequencies result in a positive *D* value. The statistics were calculated with the following parameters: *n* is the number of chromosomes, *S*_*n*_ is the number of polymorphic sites observed, *p*_*i*_ is the derived (nonancestral) allele frequency of the *i*th SNP, and *q*_*i*_ is the ancestral allele frequency of the *i*th SNP. The Tajima’s *D* score was given as:

$$ D=\frac{\pi -{\theta}_s}{\sqrt{Var\left(\pi -{\theta}_s\right)}}, $$

where

$$ \pi =\frac{n}{n-1}{\displaystyle {\sum}_{i=1}^{S_n}2{p}_i{q}_i} $$

and

$$ {\theta}_s=\frac{S_n}{{\displaystyle {\sum}_{i=1}^{n-1}\frac{1}{i}}}. $$

Negative values of Tajima’s *D* indicate an excess of rare variations, which is in line with positive selection. Positive values of Tajima’s *D*, on the other hand, suggest an excess of common variations in a region which can be consistent with balancing selection. Among them, only the top 1 % of the distribution of SNPs was considered to have the footprint of balancing selection and the middle 80 % were used as a control set for all downstream analyses (Additional file 1: Figure S1).

To test whether the increased diversity is a consequence of balancing selection acting on different alleles at a given locus, the mean pairwise π was computed within and between haplogroups that were defined by the allele that each haplotype carried at the focal SNP (Fig. 4; Additional file 1: Figure S3). For regions with evidence of a long-lived balancing selection (presence of SNPs segregating commonly in humans and chimpanzees), we calculated the mean pairwise π in humans and chimpanzees (using the phased PanMap set based on mapping to the chimpanzee reference [37]) in 500-bp sliding windows. To this end, we identified the orthologous chimpanzee positions for each human SNP by running the UCSC liftOver tool between the panTro2 and hg19 reference assemblies and selecting the coincident sets of human and chimpanzee SNPs.

To verify the signatures of Tajima’s *D*, we also performed a Hudson–Kreitman–Aguade (HKA) test in the EUR panel (Additional file 1: Figure S2a, b). The HKA test compares the level of polymorphism (within-species diversity) to the level of substitution (between-species divergence). We conducted the maximum likelihood HKA test [16] by using the MLHKA software (http://wright.eeb.utoronto.ca/programs/). The surrounding 1-kb regions of the DNase footprint SNPs were compared with 99 neutrally evolved regions that were selected as previously described [11, 38]. The number of segregating sites and pairwise number of between-species differences in each region were used as the input. Chimpanzee was used as an outgroup in this analysis. To test for selection, the program was run under a neutral model in which the number of selected loci was zero, and then under a selection model in which the surrounding 1-kb region of the focal SNP was regarded as the only selected locus. Statistical significance was assessed by the likelihood ratio test, in which twice the difference in log likelihood between the selection and neutral models approximately follows the *x*^2^ distribution with a degree of freedom 1 (number of the selected loci). To ensure the robustness of the outputs, we applied a chain length of 100,000. For each test site, the selection parameter *k* and the *P* value were obtained from the likelihood ratio test. The selection parameter *k* indicates a *k*-fold elevation to diversity over the neutral expectation at the given locus. Therefore, *k* > 1 supports balancing selection.

Selection that favors the same alleles in all populations lowers *F*_*st*_, an indicator of population differentiation [17]. Low *F*_*st*_ values can indicate alleles that are maintained at similar frequencies in different populations against the tendency for neutral drift to cause these frequencies to vary [39, 40]. The pairwise *F*_*st*_ among the three populations (ASN, EUR, and AFR) was calculated as *F*_*st*_ = 1 − *H*_*s*_/*H*_*T*_, where *H*_*S*_ denotes the average subpopulation heterozygosity and *H*_*T*_ denotes the total heterozygosity (Additional file 1: Figure S2c). It can also be used to infer adaptive evolution between populations.

Prior to the functional analysis of the identified balanced SNPs, we further excluded a few candidate SNPs that have a lower Tajima’s *D* score than that of linked nonsynonymous SNPs because of the concern that those balancing signals may have been attributed to the regulatory SNPs hitch-hiking the nonsynonymous variants that are actually subject to balancing selection (Fig. 1; Additional file 1: Figure S4). The linkage disequilibrium (LD) was calculated in each population with an r^2^ > 0.9 and a chromosomal distance <500 kb as the parameters of the SNAP proxy tool (http://www.broadinstitute.org/mpg/snap/) [41].

### Enrichment tests for balanced SNPs

To test for the enrichment of the balanced SNPs in open chromatin regions in different cell types, we obtained the whole-genome DNase hypersensitive site (DHS) datasets for 125 cell types as provided by the ENCODE Project [42] and those for 349 samples (encompassing 53 distinct tissue types) studied in the Roadmap Epigenomics Project [43]. We used these footprint data for 41 cell types [30], which were initially used to identify balanced regulatory SNPs. For an enrichment score that reflects the degree to which a set of given SNPs is overrepresented in a specific cell type, the ratio of the number of the balanced SNP overlaps to the number of the control SNP overlaps in each cell type was computed (Fig. 1b; Additional file 1: Figure S5). The full list of the tested cell types along with the enrichment test results are provided in Additional file 2. To conduct a similar analysis in TF binding regions, we obtained the chromatin immunoprecipitation sequencing (ChIP-seq) datasets for 161 different TFs from the ENCODE Project [42] (Fig. 1c; Additional file 1: Figure S6). The same enrichment analysis was performed for the TF binding regions.

### Analysis of target gene functions

To identify the genes regulated by the *cis*-regulatory region containing the SNPs of interest, we used a couple of whole-genome promoter–enhancer mapping datasets. We defined a promoter as the region 500-bp upstream of the transcription start site of a gene based on GENCODE v.19 annotation and obtained genome-wide multiple cell-type enhancer information from two previous studies. The first dataset for the distal enhancer-to-promoter connections was created by using the correlation of the sequencing tag density between distal DHSs and proximal DHSs across cell types [44]. The second dataset was developed based on the correlations between enhancer RNA levels and messenger RNA levels as measured by the Cap Analysis Gene Expression (CAGE) method across the FANTOM5 panel of ~1000 distinct cell types [45].

To determine the function of the genes that are targeted by the balanced SNPs in *cis*-regulatory regions, we conducted a gene set enrichment analysis using gene targets that were identified as described above. The hyper-geometric enrichment test was performed for each Gene Ontology (GO) term using the online tool Web-Based Gene Set Analysis Toolkit (WebGestalt) [46]. To reduce the type I error, we conducted the Benjamini–Hochberg (BH) correction for multiple testing [47]. We selected GO terms with the adjusted *P* values < 0.05 as significantly enriched function. These procedures were repeated for the set of the balanced SNPs detected in each subpopulation (Additional file 3). A total of 646 target genes mapped to 986 balanced SNPs from the EUR panel, 651 genes mapped to 910 SNPs from the ASN panel, and 1006 genes mapped to 1082 SNPs in the AFR panel were used in our GO analysis.

### Allelic imbalance and motif divergence

To test whether the balanced SNPs have regulatory consequences, we examined the differential contribution of the two alleles at each locus to overall chromatin accessibility at the region centered on the focal SNP. We screened all heterozygous variants in the 41 cell types of DNase footprint sequencing BAM files from the ENCODE Project [29]. For a more accurate variant detection, we carried out several clean-up procedures, including removal of duplicates using the Picard tools (http://broadinstitute.github.io/picard/), and performed local realignment and base quality recalibration using the Genome Analysis Tool Kit (GATK) [48]. After variant calling, GATK variant filtration was performed to retain the sites for which the map quality (MQ) was ≥30 and the Phred scaled probability that a polymorphism exists (QUAL) was ≥30. We used heterozygous variants with a minimum read depth of 10 in the footprint data and calculated the degree of allelic imbalance as the log2 ratio of the number of reads carrying each allele (Fig. 2a). To set the median of the allele imbalance score of the control SNPs to zero for the purpose of normalization, we subtracted the median of the control set of SNPs from the allelic imbalance distribution of the balanced SNPs as well as the control SNPs in each cell type. As another test for the regulatory effects of the balanced SNPs, we examined whether the alleles at each SNP result in differential TF binding. To identify TF binding motifs, we searched the TRANSFAC [49–51] and JASPAR [52–55] databases with a *P* value threshold of 10^−4^ using FIMO (http://meme-suite.org/doc/fimo.html) [56]. The motif search was performed for ±20-bp sequences containing either allele at each footprint SNP. We obtained the number of TFs that were predicted to bind specifically to one of the two alleles and the number of TFs that were associated with both alleles. For a motif divergence score, the ratio of the number of allele-specific TFs to the number of common TFs was used (Fig. 2b). Binding QTLs for five TFs in Yoruban lymphoblastoid cells [21] were used to test the allelic differentiation of the AFR balanced SNPs in TF binding (Additional file 4). The Fisher’s exact test was performed for the overlap of the QTLs with the balanced versus control SNPs.

### Gene expression association of balanced SNPs

We investigated whether the balanced SNPs affect gene expression. Such effects were first estimated with a linear regression model using the RNA sequencing data of 465 lymphoblastoid cell lines [57, 58] from the Geuvadis Consortium and the matched whole-genome genotypes retrieved from the 1000 Genomes Project [28]. We calculated the coefficient of determination (*R*^*2*^) and its *P* value from the linear regression between the genotype of the regulatory SNP and the expression level of the target gene (Fig. 2c). The Bonferroni adjustment was used to address multiple testing. The target genes were discovered using the methodologies described in the “Analysis of target gene functions” section above. In another test, we used the eQTL data for 850 samples in adipose, lymphoblastoid, and skin cells obtained from the MuTHER (Multiple Tissue Human Expression Resource) Project [59]. When a footprint SNP was not directly available in the genotyping array used in the project, we looked for the closest tag SNP within 500 kb in LD (*r*^2^ > 0.9) with the footprint SNP of interest [44] based on the EUR population. The significance of the association between the genotype of the footprint SNP or tag SNP and the expression level of the target gene was adjusted by the Bonferroni correction (Fig. 2d).

### Mathematical model of transcriptional noise in homozygotes and heterozygotes

For the concentration of an mRNA (*m*) and its cognate protein (*p*), the reaction rate theory of the Central Dogma can be written as:

1 $$ \left\{\begin{array}{l}\overset{.}{m}={\alpha}_m\kern0.15em f\left(\left[\mathrm{T}\mathrm{F}\right]\right)-{\gamma}_mm\\ {}\overset{.}{p}=\alpha {}_pm-{\gamma}_pp\end{array}\right., $$

where *α*_*m*_ is the rate of transcription, *α*_*p*_ is the rate of translation, *γ*_*m*_ is the rate of mRNA decay, and *γ*_*p*_ is the rate of protein decay. The function *f*([TF]) indicates the equilibrium concentration of the transcription initiation complex consisting of the DNA (D), RNA polymerase (RNA_p_), and transcription factor (TF). If the TF is an activator, *f*([TF]) is an increasing function. If the TF is a repressor, *f*([TF]) is a decreasing function. To find an explicit form of *f*([TF]), we impose:

- The equilibrium condition on DNA–protein binding:

2 $$ \begin{array}{c}\hfill f\left(\left[\mathrm{T}\mathrm{F}\right]\right)=\left[{\mathrm{D}}_{01}\right]+\left[{\mathrm{D}}_{11}\right]=\frac{\left[\mathrm{R}\mathrm{N}\mathrm{A}\right]\left[{\mathrm{D}}_{00}\right]}{K_1}+\frac{\left[\mathrm{R}\mathrm{N}{\mathrm{A}}_{\mathrm{p}}\right]\left[{\mathrm{D}}_{00}\right]\left[\mathrm{T}\mathrm{F}\right]}{K_2{K}_3}\hfill \\ {}\hfill =\frac{\left[\mathrm{R}\mathrm{N}{\mathrm{A}}_{\mathrm{p}}\right]\left[{\mathrm{D}}_{00}\right]}{K_1}\left(1+\frac{K_1\left[\mathrm{T}\mathrm{F}\right]}{K_2{K}_3}\right),\hfill \end{array} $$

where the dissociation constant *Ki* is defined in Fig. 3b.

- From the constancy of the gene copy number (set to 1):

3 $$ {\displaystyle \sum_{ij}\left[{\mathrm{D}}_{ij}\right]=\left[{\mathrm{D}}_{00}\right]\left\{1+\frac{\left[\mathrm{T}\mathrm{F}\right]}{K_2}+\frac{\left[\mathrm{R}\mathrm{N}{\mathrm{A}}_{\mathrm{p}}\right]}{K_1}\left(1+\frac{K_1\left[\mathrm{T}\mathrm{F}\right]}{K_2{K}_3}\right)\right\}}=1, $$

the concentration of the bare, or unbound, DNA satisfies

4 $$ {\left[{\mathrm{D}}_{00}\right]}^{-1}=1+\frac{\left[\mathrm{R}\mathrm{N}{\mathrm{A}}_{\mathrm{p}}\right]}{K_1}+\frac{\left[\mathrm{T}\mathrm{F}\right]}{K_2}\left(1+\frac{\left[\mathrm{R}\mathrm{N}{\mathrm{A}}_{\mathrm{p}}\right]}{K_3}\right)=1+R+\left(1+sR\right)T, $$

where *R*≡[RNA_p_]/*K*_1_ and *T*≡[TF]/*K*_2_ is the concentration of the RNA polymerase and TF scaled by the polymerase–DNA dissociation constant *K*_1_ and the TF–DNA dissociation constant *K*_2_, respectively. The positive and negative effect of TF action is captured by *s*≡*K*_1_/*K*_3_, which is larger than 1 for an activator while smaller than 1 for a repressor. An activator that promotes the recruitment of RNA_p_ (*K*_3_ < *K*_1_) leads to *s* > 1 and *φ* > 1, whereas for a repressor, the inequalities point in the opposite direction. Rearranging the terms, we obtain [60]:

5 $$ f\left(\mathrm{T}\right)=\frac{R\left(1+sT\right)}{1+R+\left(1+sR\right)T}=\frac{1}{1+{R}^{-1}}\left[1+\frac{\left(s-{\tau}^{-1}\right)T}{1+T/\tau}\right]=f(0)\left(1+\frac{\phi -1}{1+\tau /T}\right), $$

where *τ* = (1 + *R*)/(1 + *sR*), *ϕ* = *f*(∞)/*f*(0) = (*R* + 1)/(*R* + *S*^− 1^) is the fold change, and *f*(0) represents a value corresponding to the basal level of gene expression.

For the cells heterozygous for the given *cis*-regulatory sequences, where the binding equilibrium between the TF and its cognate binding site is controlled by two distinct equilibrium (dissociation) constants *K*_2_ and *λK*_2_ for the two alleles, the rate equation Eq. 1 can be generalized to:

6 $$ \left\{\begin{array}{l}\overset{.}{m}=\frac{\alpha m}{2}\left\{f(T)+f\left({\lambda}^{-1}T\right)\right\}-{\gamma}_mm\\ {}\overset{.}{p}={\alpha}_pm-{\gamma}_pp\end{array}\right.. $$

The corresponding homozygous cells under consideration have a pair of identical regulatory sequences with $\overline{\lambda}{K}_2$. For a fair comparison of noise levels between heterozygous and homozygous gene expression, we require the same mean expression level. That is, from Eq. 5:

7 $$ \left\langle \frac{1}{1+\tau /T}+\frac{1}{1+\lambda \tau /T}\right\rangle =\left\langle \frac{2}{1+\overline{\lambda}\tau /T}\right\rangle, $$

where the angled brackets denote the statistical ensemble average. For a gamma-distributed concentration of the TF [61] with the shape and scale parameters *a* and *b*, that is [TF] ~ Γ(*a*,*b*), the rescaled random variable *z*[TF] also follows the gamma distribution as Γ(*a*, *zb*).

Now we consider the fluctuations, particularly in [TF]. The aim is to compare protein noise levels between a homozygote with the normalized transcription rate ${f}_{\overline{\lambda}}(T)=f\left(T/\overline{\lambda}\right)$ and a heterozygote with $\mathit{\mathsf{g}}\lambda (T)=\frac{1}{2}\left[f(T)+f\left(T/\lambda \right)\right]$ that have the same level of average gene expression. With that, the variance in mRNA and protein levels is given by:

8a $$ {\sigma}_m^2=\frac{\alpha_m\left\langle \mathit{\mathsf{g}}\right\rangle }{\gamma_m}+{\left(\frac{\alpha_m{\sigma}_{\mathit{\mathsf{g}}}}{\gamma_m}\right)}^2=\left\langle m\right\rangle \left[1+\left\langle m\right\rangle {\left(\frac{\sigma_{\mathit{\mathsf{g}}}}{\left\langle \mathit{\mathsf{g}}\right\rangle}\right)}^2\right], $$

8b $$ {\sigma}_p^2=\left\langle p\right\rangle \left(1+\frac{\left\langle p\right\rangle }{\left\langle m\right\rangle}\frac{1}{1+{\gamma}_m/{\gamma}_p}+\frac{\left\langle p\right\rangle }{{\left\langle m\right\rangle}^2}{\sigma}_m^2\right), $$

where 〈 ⋅ 〉 is the expectation over the statistical ensemble. Thus, the noise level defined as the squared coefficient of variation:

9a $$ {\eta}_m^2\equiv \frac{\sigma_m^2}{{\left\langle m\right\rangle}^2}=\frac{1}{\left\langle m\right\rangle }+{\eta}_{\mathit{\mathsf{g}}}^2, $$

9b $$ {\eta}_p^2\equiv \frac{\sigma_p^2}{{\left\langle p\right\rangle}^2}=\frac{1}{\left\langle p\right\rangle }+\frac{1}{\left\langle m\right\rangle}\frac{1}{1+{\gamma}_m/{\gamma}_p}+{\eta}_m^2 $$

gives a fractional measure of the stochastic fluctuations. Note that the first term in *η*_*p*_^2^ (Eq. 9b), which scales with the inverse of the mean protein level, is reminiscent of a Poisson process and indicates (i) the so-called intrinsic noise originating from random births and deaths of the individual protein molecule. Additional terms reflect an extrinsic noise stemming from (ii) the mRNA noise at a given strength of TF–DNA binding and from (iii) the allele-dependent variation of TF-binding affinity, which is contained in ${\eta}_{\mathsf{g}}^2$ .

In a steady state, where $\left\langle p\right\rangle =\frac{\alpha_p}{\gamma_p}\left\langle m\right\rangle =\frac{\alpha_m{\alpha}_p}{\gamma_m{\gamma}_p}\left\langle {\mathit{\mathsf{g}}}_{\lambda }(T)\right\rangle$, the protein noise can be additively decomposed as:

10 $$ {\eta}_p^2\left[{\mathit{\mathsf{g}}}_{\lambda }(T)\right]=\frac{\gamma_m}{\alpha_m}\frac{1}{\left\langle {\mathit{\mathsf{g}}}_{\lambda }(T)\right\rangle}\left(1+\frac{\gamma_p}{\alpha_p}+\frac{1}{1+{\gamma}_m/{\gamma}_p}\right)+{\eta}_{\mathit{\mathsf{g}}}^2, $$

where the zygosity comes into play only through ${\eta}_{\mathit{\mathsf{g}}}^2$. To calculate the noise level in a homozygote with the normalized transcription rate $f\left(T/\overline{\lambda}\right)\equiv {f}_{\overline{\lambda}}(T)$, we first determine $\overline{\lambda}$, as a function of λ, that satisfies $\left\langle {\mathit{\mathsf{g}}}_{\lambda }(T)\right\rangle =\left\langle {f}_{\overline{\lambda}}(T)\right\rangle$ from the requirement of Eq. 7. Thus, we obtained the difference in the noise level, $\Delta {\eta}^2\equiv {\eta}_p^2\left[{f}_{\overline{\lambda}}(T)\right]-{\eta}_p^2\left[\mathit{\mathsf{g}}\lambda (T)\right]$, as a function of *λ* or other parameters across a range of in vivo biochemical parameters [25, 26, 62]. Figure 3c shows ∆*η*^2^ as a function of *λ*.

### Expression and chromatin noise from single cell data

We employed RNA sequencing results for 62 single cells from the GM12878 cell line [63]. This cell line was derived from the NA12878 sample, for which a fully phased genome sequence is available [28]. The coefficient of variation, measured as the standard deviation divided by the mean ($CV=\frac{\sigma }{\mu }$), is the most direct and unambiguous measure of gene expression noise [64]. We calculated the *CV* of the read counts normalized across the samples. To work only with genes with uniform power to detect high or low variance, we excluded transcripts with an average FPKM <100 and hence a saturated *CV* [65]. We fitted the curve using the parameterization $C{V}^2={a}_0+\frac{a_1}{\mu }$ to capture the dependence of the *CV*^2^ on *μ* [65] (Fig. 3e). We differentiated homozygous and heterozygous loci using the GM12878 genotypes available from the 1000 Genomes Project [28] and discovered target genes based on the GM12878-specific enhancer–promoter maps created by the Chromatin Interaction Analysis by Paired-End Tag sequencing (ChIA-PET) [66], chromosome conformation capture technology (Capture Hi-C) [67], and Integrated Methods for Predicting Enhancer Targets (IM-PET) [68]. ChIA-PET interactions with four or more PET counts were used. We ran HOMER (http://homer.salk.edu/homer/ngs/) to identify significant (*P* < 10^-6^) promoter–capture Hi-C interactions. The IM-PET method was used for our main analyses. We used the combination of the two experimental datasets (ChIA-PET and Capture Hi-C) to confirm that the observed expression noise-selection relationships are not dependent on enhancer–promoter mapping data (Additional file 1: Figure S10).

To analyze cell-to-cell variation in chromatin accessibility, we used the assay for transposase-accessible chromatin sequencing (ATAC-seq) data set for 254 individual cells from the GM12878 cell line [69]. This dataset was downloaded from the NCBI Gene Expression Omnibus (GEO) under accession number GSE65360. We modified and ran the analysis scripts provided by the authors. We used as input the chromosomal coordinates of chromatin accessibility peaks (top 50,000 non-overlapping 500-bp summits) and various features associated with each peak, including fragment counts and sequence bias scores. For our analysis, we assigned the Tajima’s *D* score and heterozygosity in GM12878 to each accessible chromatin peak. We computed the aggregate measure of cell-to-cell variability in chromatin accessibility for peaks falling within a given range of Tajima’s *D*. The standard deviation of the calculated variability was obtained by bootstrapping cells as previously described [69] (Fig. 3f). For a gene-wise aggregate measure of chromatin accessibility noise, the average of the *cis*-regulatory regions connected to the same gene was obtained by interrogating the IM-PET-based GM12878 enhancer–promoter map [68].

### Analysis of gene expression level and transcriptional responsiveness

To test whether the advantage of the selected SNPs lies in flexibility of transcriptional responses to various external signals, we sought to analyze gene expression data for the human lymphoblastoid cell line GM12878. This cell line was derived from the NA12878 sample, for which a fully phased genome sequence is available [28]. For the first dataset, we downloaded the data for gene expression changes in response to doxorubicin treatment from the NCBI GEO under accession number GSE51709 [70]. The raw expression measures were treated with the Affymetrix Expression Console using the gene-level RMA summarization and sketch-quantile normalization methods. The second gene expression dataset was downloaded from the GEO under accession number GSE26835. In this dataset, the expression measures were obtained prior to ionizing radiation and at 2 and 6 h after exposure to 10 Gy of ionizing radiation [71]. This dataset was created with the Affymetrix Expression Console using the MAS5 probe summary method and global scaling normalization method. For each gene, the degree of transcriptional responsiveness was calculated as the maximum absolute gene expression change in response to the treatment among different time points or samples. To link the heterozygosity and selection strengths of each regulatory SNP with the transcriptional response of its target gene, we used the GM12878-specific enhancer–promoter maps generated by the IM-PET [68] methods. The degree of gene expression changes in response to doxorubicin or ionizing radiation was computed for the control SNPs (corresponding to the middle 80 % of the Tajima’s *D* distribution), homozygous SNPs, and heterozygous SNPs with varying Tajima’s *D* or HKA *k* (Additional file 1: Figure S11a, b). The degree of chromatin changes in response to TNF-α treatment was calculated for the same groups of footprint SNPs. We used the ATAC-seq data set for GM12878 single cells [69] described above (Additional file 1: Figure S11c). The aggregate measure of chromatin accessibility across the single cells was obtained for each chromatin peak.

We sought to test whether the balanced SNPs are advantageous when their target genes are highly expressed, in which case extrinsic noise is a dominant source of cell-to-cell variability. We chose white blood cells for which mRNA sequencing data were available from the Roadmap Epigenomics Project, namely, CD4 naïve primary cells, CD4 memory primary cells, CD8 naïve primary cells, and peripheral blood mononuclear primary cells. The IM-PET-based enhancer–promoter map for CD4 naïve T cells [68] was used to associate the Tajima’s *D* score of the regulatory SNPs with the expression level of the target genes calculated as FPKM. Additionally, we used the two Affymetrix datasets used for the above responsiveness tests, namely, the doxorubicin response data (GSE51709) and the radiation response data (GSE26835). We performed sample-wise normalization, in which the mean expression level of all genes in each sample (CD4 naïve primary cells, CD4 memory primary cells, CD8 naïve primary cells, peripheral blood mononuclear primary cells, GM12878 before doxorubicin treatment, GM12878 after doxorubicin treatment, GM12878 before irradiation, and GM12878 after irradiation) was set to zero.

## Additional files

Additional file 1:
**Figure S1.** **a**–**c** The distribution of Tajima’s D for DNase footprint SNPs in each subpopulation. **d** Overlapping of the SNPs with the largest (top 1 %) positive *D* from each subpopulation. **Figure S2.** **a** The distribution of HKA *k* values. **b** HKA *P* values as afunction of HKA *k* values. **c** The distribution of Fst values. **Figure S3.** Divergence between haplogroups compared with within haplogroups in a EUR, **b** ASN, and **c** AFR. **Figure S4.** Tajima’s *D* of the selected (top 1 %) DNase footprint SNPs and non-synonymous SNPs that are in linkage disequilibrium with them in **a** EUR, **b** ASN, and **c** AFR. **Figure S5.** Enrichment of the selected footprint SNPs in *cis*-regulatory regions of different cell types in EUR, ASN, and AFR. **Figure S6.** **a**, **b** Enrichment of the selected footprint SNPs in the binding regions of different TFs. **c**–**e** Zoom in for the top TFs. **Figure S7.** The localization of the footprint SNPs and linked non-synonymous SNPs at the clusters of the **a** MHC class and **b**, **c** SPRR and LCE genes. **Figure S8.** **a** Redrawing of Fig. 3e using HKA *k* instead of Tajima’s *D*. **b** Redrawing of Fig. 3f using HKA *k* instead of Tajima’s *D*. **Figure S9.** Negative correlation between the degree of balancing selection signature and the noise strength. **Figure S10.** **a** Redrawing of Fig. 3e using the ChIA-PET and Capture Hi-C data instead of the IM-PET data. **b** Redrawing of the left panel of **Figure S9** using the ChIA-PET and Capture Hi-C data instead of the IM-PET data. **Figure S11.** **a**, **b** The degree of gene expression changes in response to **a** doxorubicin or **b** ionizing radiation as a function of selection strength. **c** The degree of chromatin changes inresponse to TNF-α treatment as a function of balancing selection signature. (PDF 3 kb) Additional file 2: Details of enrichment tests for the selected footprint SNPs in *cis*-regulatory regions of different cell types (Fig. 1b; Additional file 1: Figure S5). (XLSX 54 kb) Additional file 3: GO categories enriched in genes associated with the selected SNPs. (XLSX 18 kb) Additional file 4: Enrichment of the selected SNPs for TF binding QTLs. (XLSX 9 kb)

## References

[1] Pasvol G, Weatherall D, Wilson R (1978). Cellular mechanism for the protective effect of haemoglobin S against P. falciparum malaria. Nature.

[2] Roth E, Friedman M, Ueda Y, Tellez I, Trager W, Nagel R (1978). Sickling rates of human AS red cells infected in vitro with Plasmodium falciparum malaria. Science..

[3] Schroeder SA, Gaughan DM, Swift M (1995). Protection against bronchial asthma by CFTR delta F508 mutation: a heterozygote advantage in cystic fibrosis. Nat Med..

[4] Hughes AL, Nei M (1988). Pattern of nucleotide substitution at major histocompatibility complex class I loci reveals overdominant selection. Nature..

[5] Penn DJ, Damjanovich K, Potts WK (2002). MHC heterozygosity confers a selective advantage against multiple-strain infections. Proc Natl Acad Sci U S A..

[6] Arking DE, Krebsova A, Macek M, Arking A, Mian IS, Fried L (2002). Association of human aging with a functional variant of klotho. Proc Natl Acad Sci U S A..

[7] Dubal DB, Yokoyama JS, Zhu L, Broestl L, Worden K, Wang D (2014). Life extension factor klotho enhances cognition. Cell Rep..

[8] Savova V, Chun S, Sohail M, McCole RB, Witwicki R, Gai L (2016). Genes with monoallelic expression contribute disproportionately to genetic diversity in humans. Nat Genet..

[9] Chess A (2012). Mechanisms and consequences of widespread random monoallelic expression. Nat. Rev. Genet..

[10] Leffler EM, Gao Z, Pfeifer S, Ségurel L, Auton A, Venn O (2013). Multiple instances of ancient balancing selection shared between humans and chimpanzees. Science..

[11] Gokcumen O, Zhu Q, Mulder LCF, Iskow RC, Austermann C, Scharer CD (2013). Balancing selection on a regulatory region exhibiting ancient variation that predates human-neandertal divergence. PLoS Genet..

[12] Cagliani R, Fumagalli M, Riva S, Pozzoli U, Comi GP, Menozzi G (2008). The signature of long-standing balancing selection at the human defensin beta-1 promoter. Genome Biol..

[13] Sellis D, Callahan BJ, Petrov DA, Messer PW (2011). Heterozygote advantage as a natural consequence of adaptation in diploids. Proc Natl Acad Sci U S A..

[14] Tajima F (1989). Statistical method for testing the neutral mutation hypothesis by DNA polymorphism. Genetics..

[15] Hudson RR, Kreitman M, Aguade M (1987). A test of neutral molecular evolution based on nucleotide data. Genetics..

[16] Wright SI, Charlesworth B (2004). The HKA test revisited: a maximum-likelihood-ratio test of the standard neutral model. Genetics..

[17] Weir BS, Cockerham CC (1984). Estimating F-statistics for the analysis of population structure. Evolution (N Y).

[18] Charlesworth D (2006). Balancing selection and its effects on sequences in nearby genome regions. PLoS Genet..

[19] Cabral A, Voskamp P, Cleton-Jansen AM, South A, Nizetic D, Backendorf C (2001). Structural organization and regulation of the small proline-rich family of cornified envelope precursors suggest a role in adaptive barrier function. J Biol Chem..

[20] Jackson B, Tilli CMLJ, Hardman MJ, Avilion A, MacLeod MC, Ashcroft GS (2005). Late cornified envelope family in differentiating epithelia--response to calcium and ultraviolet irradiation. J Invest Dermatol..

[21] Tehranchi AK, Myrthil M, Martin T, Hie BL, Golan D, Fraser HB (2016). Pooled ChIP-Seq links variation in transcription factor binding to complex disease risk. Cell..

[22] Lehner B (2008). Selection to minimise noise in living systems and its implications for the evolution of gene expression. Mol Syst Biol..

[23] Metzger BPH, Yuan DC, Gruber JD, Duveau F, Wittkopp PJ (2015). Selection on noise constrains variation in a eukaryotic promoter. Nature..

[24] Volfson D, Marciniak J, Blake WJ, Ostroff N, Tsimring LS, Hasty J (2006). Origins of extrinsic variability in eukaryotic gene expression. Nature..

[25] Taniguchi Y, Choi PJ, Li G-W, Chen H, Babu M, Hearn J (2010). Quantifying E. coli proteome and transcriptome with single-molecule sensitivity in single cells. Science.

[26] Stewart-Ornstein J, Weissman JS, El-Samad H (2012). Cellular noise regulons underlie fluctuations in Saccharomyces cerevisiae. Mol Cell..

[27] Kambayashi T, Laufer TM (2014). Atypical MHC class II-expressing antigen-presenting cells: can anything replace a dendritic cell?. Nat Rev Immunol..

[28] The 1000 Genomes Project Consortium (2012). An integrated map of genetic variation from 1,092 human genomes. Nature.

[29] Bernstein BE, Birney E, Dunham I, Green ED, Gunter C, Snyder M (2012). An integrated encyclopedia of DNA elements in the human genome. Nature..

[30] Neph S, Vierstra J, Stergachis AB, Reynolds AP, Haugen E, Vernot B (2012). An expansive human regulatory lexicon encoded in transcription factor footprints. Nature..

[31] Pickrell JK, Gaffney DJ, Gilad Y, Pritchard JK (2011). False positive peaks in ChIP-seq and other sequencing-based functional assays caused by unannotated high copy number regions. Bioinformatics..

[32] Derrien T, Estellé J, Marco Sola S, Knowles DG, Raineri E, Guigó R (2012). Fast computation and applications of genome mappability. PLoS One..

[33] Bailey JA, Gu Z, Clark RA, Reinert K, Samonte RV, Schwartz S (2002). Recent segmental duplications in the human genome. Science..

[34] Bailey JA, Yavor AM, Massa HF, Trask BJ, Eichler EE (2001). Segmental duplications: organization and impact within the current human genome project assembly. Genome Res..

[35] Wigginton JE, Cutler DJ, Abecasis GR (2005). A note on exact tests of Hardy-Weinberg equilibrium. Am J Hum Genet..

[36] DeGiorgio M, Lohmueller KE, Nielsen R (2014). A model-based approach for identifying signatures of ancient balancing selection in genetic data. PLoS Genet..

[37] Auton A, Fledel-Alon A, Pfeifer S, Venn O, Ségurel L, Street T (2012). A fine-scale chimpanzee genetic map from population sequencing. Science..

[38] Fumagalli M, Cagliani R, Pozzoli U, Riva S, Comi GP, Menozzi G (2009). Widespread balancing selection and pathogen-driven selection at blood group antigen genes. Genome Res..

[39] Schierup MH, Vekemans X, Charlesworth D (2000). The effect of subdivision on variation at multi-allelic loci under balancing selection. Genet Res..

[40] Beaumont MA, Balding DJ (2004). Identifying adaptive genetic divergence among populations from genome scans. Mol Ecol..

[41] Johnson AD, Handsaker RE, Pulit SL, Nizzari MM, O’Donnell CJ, de Bakker PI (2008). SNAP: a web-based tool for identification and annotation of proxy SNPs using HapMap. Bioinformatics..

[42] Thurman RE, Rynes E, Humbert R, Vierstra J, Maurano MT, Haugen E (2012). The accessible chromatin landscape of the human genome. Nature..

[43] Consortium RE, Kundaje A, Meuleman W, Ernst J, Bilenky M, Yen A (2015). Integrative analysis of 111 reference human epigenomes. Nature..

[44] Maurano MT, Humbert R, Rynes E, Thurman RE, Haugen E, Wang H (2012). Systematic localization of common disease-associated variation in regulatorty DNA. Science..

[45] Andersson R, Gebhard C, Miguel-Escalada I, Hoof I, Bornholdt J, Boyd M (2014). An atlas of active enhancers across human cell types and tissues. Nature..

[46] Wang J, Duncan D, Shi Z, Zhang B (2013). WEB-based GEne SeT AnaLysis Toolkit (WebGestalt): update 2013. Nucleic Acids Res..

[47] Benjamini Y, Hochberg Y (1995). Controlling the false discovery rate: a practical and powerful approach to multiple testing. J R Stat Soc Ser B..

[48] McKenna A, Hanna M, Banks E, Sivachenko A, Cibulskis K, Kernytsky A (2010). The Genome Analysis Toolkit: a MapReduce framework for analyzing next-generation DNA sequencing data. Genome Res..

[49] Matys V, Kel-Margoulis OV, Fricke E, Liebich I, Land S, Barre-Dirrie A (2006). TRANSFAC and its module TRANSCompel: transcriptional gene regulation in eukaryotes. Nucleic Acids Res..

[50] Matys V, Fricke E, Geffers R, Gössling E, Haubrock M, Hehl R (2003). TRANSFAC: transcriptional regulation, from patterns to profiles. Nucleic Acids Res..

[51] Wingender E, Chen X, Hehl R, Karas H, Liebich I, Matys V (2000). TRANSFAC: an integrated system for gene expression regulation. Nucleic Acids Res..

[52] Bryne JC, Valen E, Tang M-HE, Marstrand T, Winther O, da Piedade I (2008). JASPAR, the open access database of transcription factor-binding profiles: new content and tools in the 2008 update. Nucleic Acids Res..

[53] Vlieghe D, Sandelin A, De Bleser PJ, Vleminckx K, Wasserman WW, van Roy F (2006). A new generation of JASPAR, the open-access repository for transcription factor binding site profiles. Nucleic Acids Res..

[54] Sandelin A, Alkema W, Engström P, Wasserman WW, Lenhard B (2004). JASPAR: an open-access database for eukaryotic transcription factor binding profiles. Nucleic Acids Res..

[55] Mathelier A, Zhao X, Zhang AW, Parcy F, Worsley-Hunt R, Arenillas DJ (2014). JASPAR 2014: an extensively expanded and updated open-access database of transcription factor binding profiles. Nucleic Acids Res..

[56] Grant CE, Bailey TL, Noble WS (2011). FIMO: scanning for occurrences of a given motif. Bioinformatics..

[57] Lappalainen T, Sammeth M, Friedländer MR, ’t Hoen PC, Monlong J, Rivas M (2013). Transcriptome and genome sequencing uncovers functional variation in humans. Nature.

[58] ’t Hoen PC, Friedländer MR, Almlöf J, Sammeth M, Pulyakhina I, Anvar SY (2013). Reproducibility of high-throughput mRNA and small RNA sequencing across laboratories. Nat Biotechnol.

[59] Grundberg E, Small KS, Hedman ÅK, Nica AC, Buil A, Keildson S (2012). Mapping cis- and trans-regulatory effects across multiple tissues in twins. Nat Genet..

[60] Ghim C-M, Almaas E (2009). Two-component genetic switch as a synthetic module with tunable stability. Phys Rev Lett..

[61] Friedman N, Cai L, Xie XS (2006). Linking stochastic dynamics to population distribution: an analytical framework of gene expression. Phys Rev Lett..

[62] Paulsson J (2004). Summing up the noise in gene networks. Nature..

[63] Marinov GK, Williams B, McCue K, Schroth GP, Gertz J, Myers RM (2014). From single-cell to cell-pool transcriptomes: stochasticity in gene expression and RNA splicing. Genome Res..

[64] Kaern M, Elston TC, Blake WJ, Collins JJ (2005). Stochasticity in gene expression: from theories to phenotypes. Nat Rev Genet..

[65] Brennecke P, Anders S, Kim JK, Kołodziejczyk A, Zhang X, Proserpio V (2013). Accounting for technical noise in single-cell RNA-seq experiments. Nat. Methods..

[66] Tang Z, Luo OJ, Li X, Zheng M, Zhu JJ, Szalaj P (2015). CTCF-mediated human 3D genome architecture reveals chromatin topology for transcription. Cell..

[67] Martin P, McGovern A, Orozco G, Duffus K, Yarwood A, Schoenfelder S (2015). Capture Hi-C reveals novel candidate genes and complex long-range interactions with related autoimmune risk loci. Nat Commun..

[68] He B, Chen C, Teng L, Tan K (2014). Global view of enhancer-promoter interactome in human cells. Proc Natl Acad Sci U S A..

[69] Buenrostro JD, Wu B, Litzenburger UM, Ruff D, Gonzales ML, Snyder MP, et al. Single-cell chromatin accessibility reveals principles of regulatory variation. Nature. 2015;523:486–90. 10.1038/nature14590 PMC4685948 26083756

[70] Su D, Wang X, Campbell MR, Song L, Safi A, Crawford GE (2015). Interactions of chromatin context, binding site sequence content, and sequence evolution in stress-induced p53 occupancy and transactivation. PLoS Genet..

[71] Smirnov D, Brady L, Halasa K, Morley M, Solomon S, Cheung VG (2012). Genetic variation in radiation-induced cell death. Genome Res..

