# Supplementary material for: Selected heterozygosity at cis-regulatory sequences increases the expression homogeneity of a cell population in humans
Source: Genome Biol. 2016 Jul 28;17:164. doi: 10.1186/s13059-016-1027-8 (PMC4964047; doi:10.1186/s13059-016-1027-8)
Supplement: Additional file 1: — Figure S1. a–c The distribution of Tajima’s D for DNase footprint SNPs in each subpopulation. d Overlapping of the SNPs with the largest (top 1 %) positive D from each subpopulation. Figure S2. a The distribution of HKA k values. b HKA P values as afunction of HKA k values. c The distribution of Fst values. Figure S3. Divergence between haplogroups compared with within haplogroups in a EUR, b ASN, and c AFR. Figure S4. Tajima’s D of the selected (top 1 %) DNase footprint SNPs and non-synonymous SNPs that are in linkage disequilibrium with them in a EUR, b ASN, and c AFR. Figure S5. Enrichment of the selected footprint SNPs in cis-regulatory regions of different cell types in EUR, ASN, and AFR. Figure S6. a, b Enrichment of the selected footprint SNPs in the binding regions of different TFs. c–e Zoom in for the top TFs. Figure S7. The localization of the footprint SNPs and linked non-synonymous SNPs at the clusters of the a MHC class and b, c SPRR and LCE genes. Figure S8. a Redrawing of Fig. 3e using HKA k instead of Tajima’s D. b Redrawing of Fig. 3f using HKA k instead of Tajima’s D. Figure S9. Negative correlation between the degree of balancing selection signature and the noise strength. Figure S10. a Redrawing of Fig. 3e using the ChIA-PET and Capture Hi-C data instead of the IM-PET data. b Redrawing of the left panel of Figure S9 using the ChIA-PET and Capture Hi-C data instead of the IM-PET data. Figure S11. a, b The degree of gene expression changes in response to a doxorubicin or b ionizing radiation as a function of selection strength. c The degree of chromatin changes inresponse to TNF-α treatment as a function of balancing selection signature. (PDF 3 kb) [file 13059_2016_1027_MOESM1_ESM.pdf]

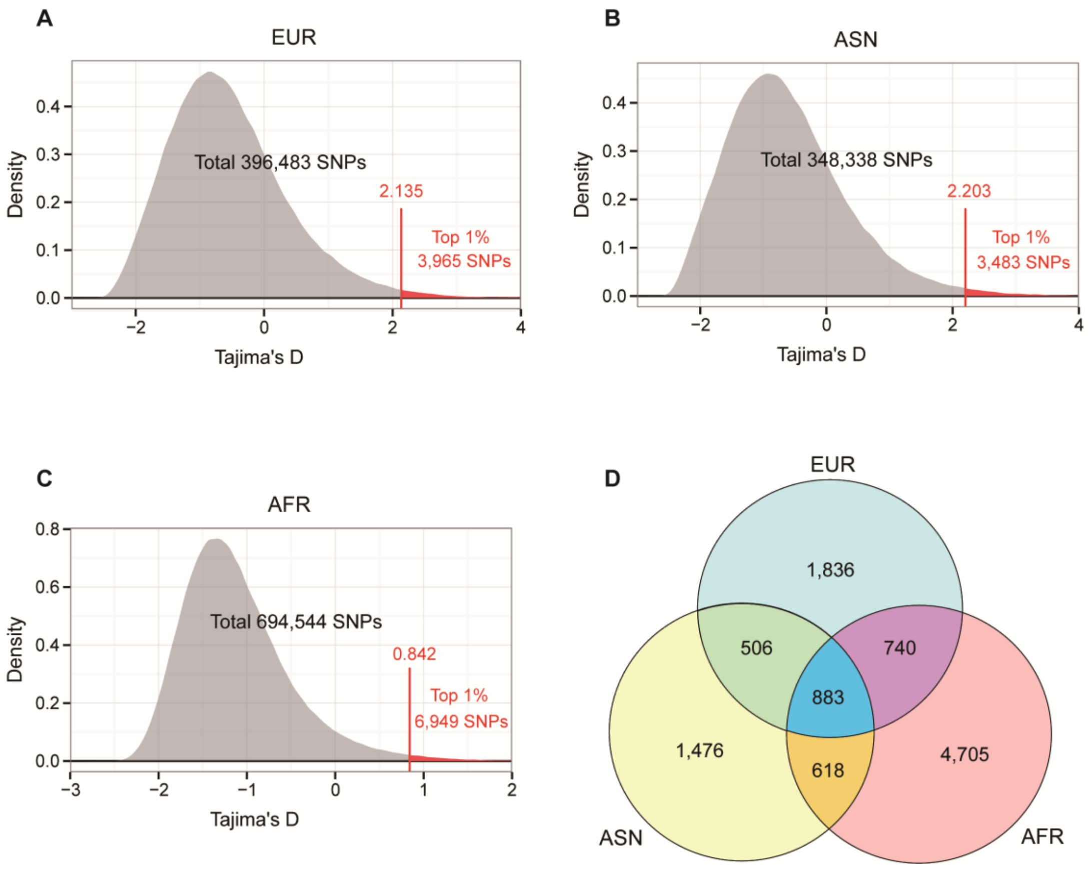

Figure S1. Distribution of Tajima's D for DNase footprints in three subpopulations. (A-C) Density plots of Tajima's D for the (A) EUR, (B) ASN, and (C) AFR panel of the 1000 genomes project, respectively. The top 1% of the distribution of the SNPs was considered to have a footprint of balancing selection and highlighted with red colour. (D) The overlaps of the top 1% SNPs identified for each population.

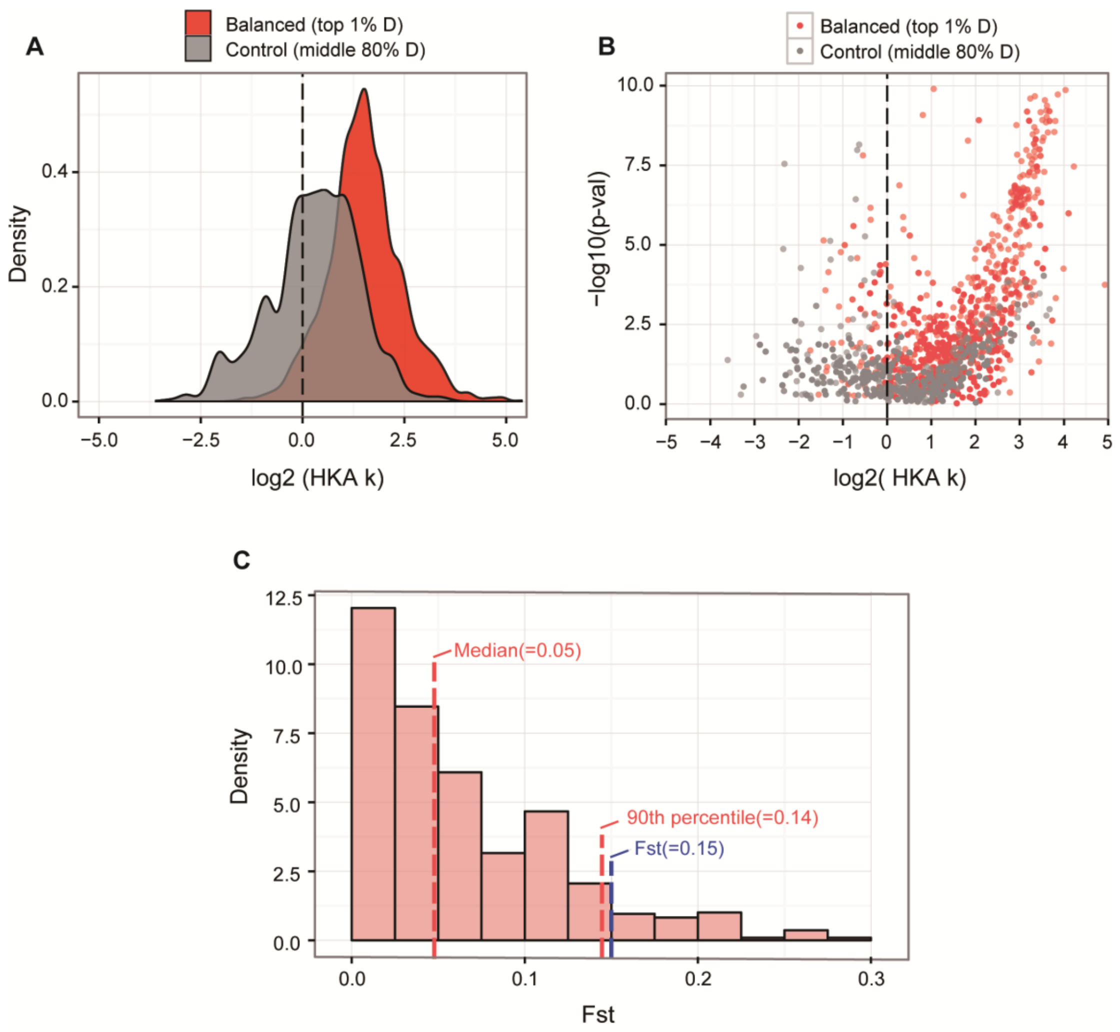

Figure S2. Comparison of Tajima's D with HKA and Fst. (A) The distribution of HKA k in a log2 scale for the top 1% D SNPs (red) and middle 80% D SNPs as control (grey) in the EUR panel. The selected SNPs showed significantly higher HKA k (95.42 % having k > 1) than the control set. (B) Volcano plot showing the P value of the HKA test plotted against the HKA k score for the balanced versus control SNPs. Data points highlighted in red (balanced SNPs with top 1% D) showed a positively skewed distribution with lower P values. (C) Frequency histogram of the Fst score. Most balanced SNPs showed low Fst values (91.2% having Fst < 0.15). The value of 0.15 is a typical estimate for neutral loci among continental populations. Outlier scores with Fst > 0.3 were not represented in the histogram.

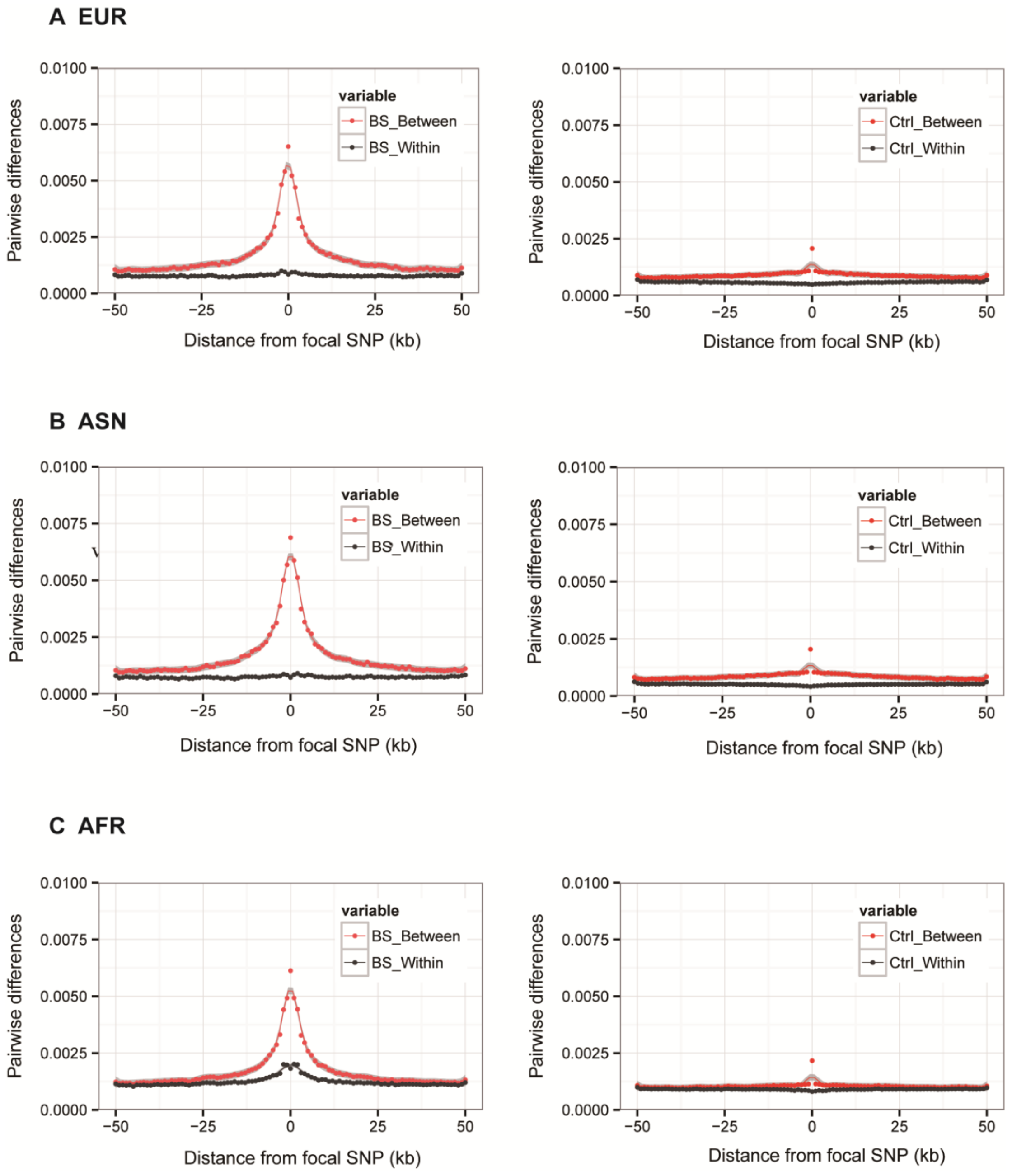

Figure S3. High divergence between haplogroups compared to within haplogroup. The mean pairwise differences of the sequences between haplogroups (red) and within haplogroup (grey) were plotted for the balanced SNPs (left) and control SNPs (right) in the (A) EUR, (B) ASN, and (C) AFR panel, respectively. The pairwise differences were calculated in 1.5-kb sliding windows and averaged for all focal SNPs.

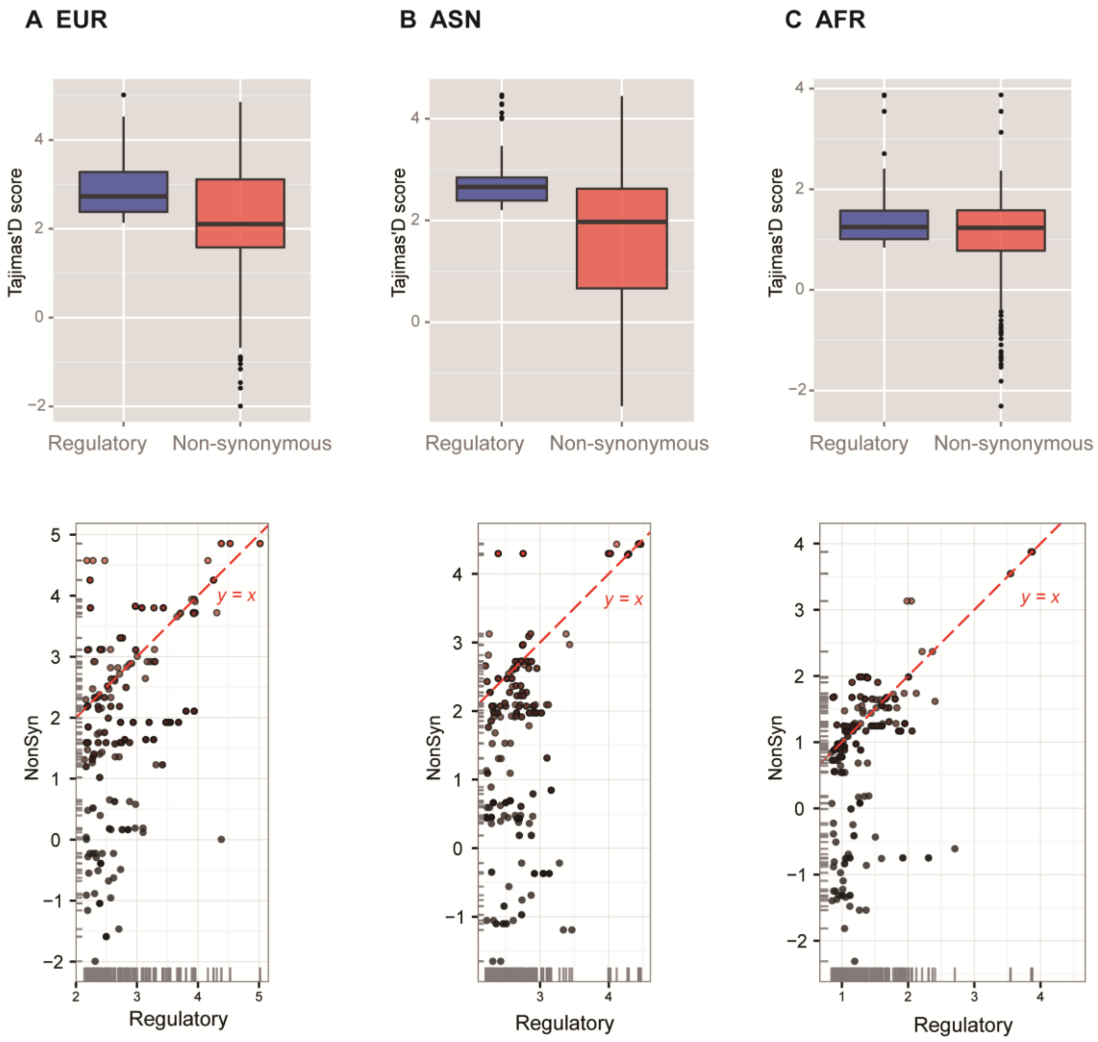

Figure S4. Comparison of Tajima's D between the selected regulatory and linked non-synonymous SNPs. Box plots (upper) and scatter plots (lower) of the Tajima's D scores for the balanced SNPs in *cis*-regulatory regions and their linked non-synonymous SNPs in linkage disequilibrium ($r^2 = 0.9$) in the (A) EUR, (B) ASN, and (C) AFR panel, respectively. Among the linked non-synonymous SNPs of each regulatory SNP, we selected that has the highest Tajima's D in the linkage disequilibrium block. One unit on the x-axis is the same length as one unit on the y-axis.

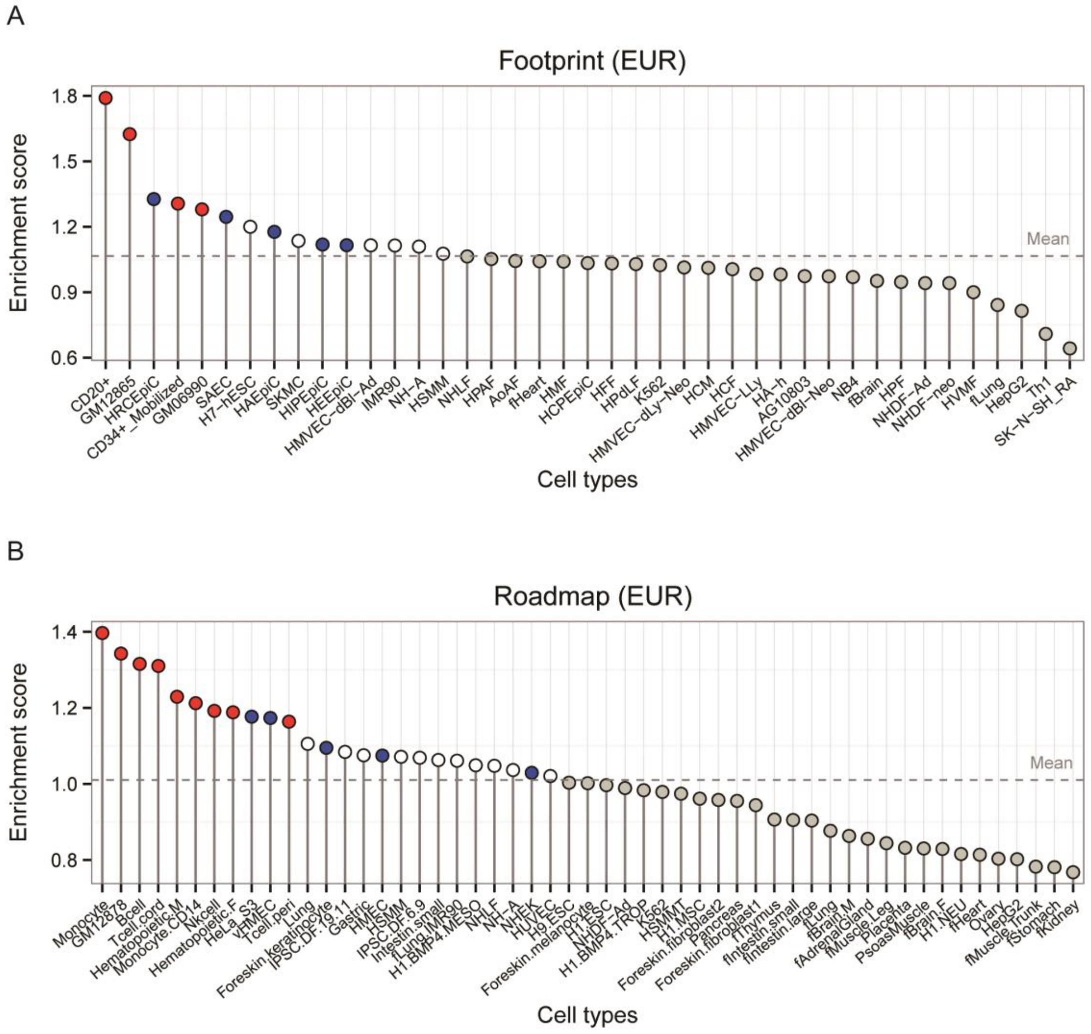

Figure S5. Enrichment of the balanced SNPs in DNase footprints. Cleveland's dot plots showing enrichment of the balanced SNPs in the *cis*-regulatory regions of diverse cell types including those for which DNase footprints are available and those used in the ENCODE project and Epigenome Roadmap project in the (A-B) EUR, (C-E) ASN, and (F-H) AFR panel. The EUR ENCODE data is provided in Fig. 1B.

C

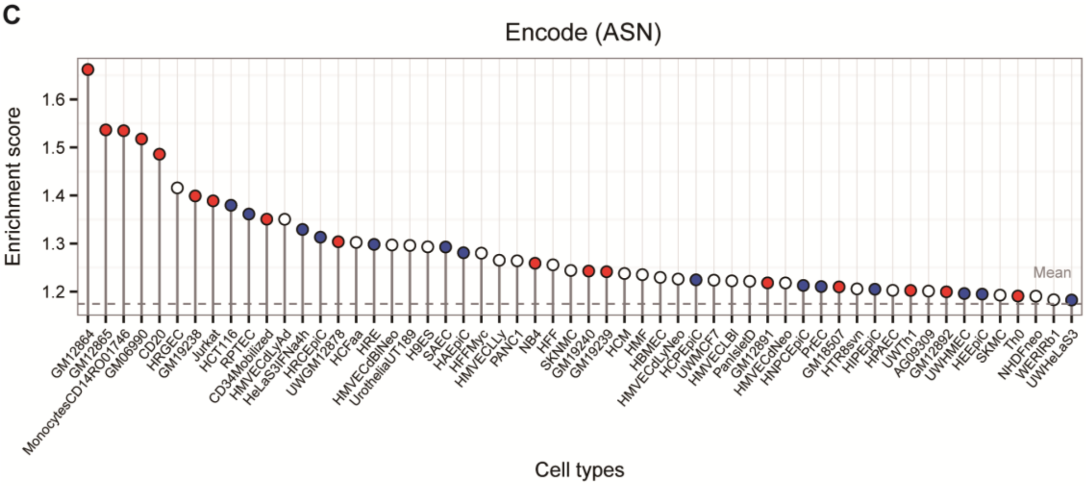

Encode (ASN)
Enrichment score
1.6
1.5
1.4
1.3
1.2
Mean
Cell types
GM12864
GM12865
MonocytesCD14RO01746
GM06990
CD20
HRGEC
GM19238
Jurkat
HCT116
RPTEC
CD34Mobilized
HMVECdLyAd
HeLaS3IFNa4h
HRCEpiC
UWGM12878
HCFaa
HRE
HMVECdBlNeo
UrothellaUT189
H9ES
SAEC
HAEpiC
HFFMyc
HMVECLLy
PANC1
NB4
HFF
SKNMC
GM19240
GM19239
HCM
HMF
HBMEC
HMVECdLyNeo
HCPEpiC
UWMCF7
HMVECLBl
PanIsletD
GM12891
HMVECdNeo
HNPCEpiC
PrEC
GM18507
HTR8svn
HIPEpiC
HPAEC
UWTh1
AG09309
GM12892
UWHMEC
HEEpiC
SKMC
Th0
NHDFneo
WERIRb1
UWHeLaS3

D

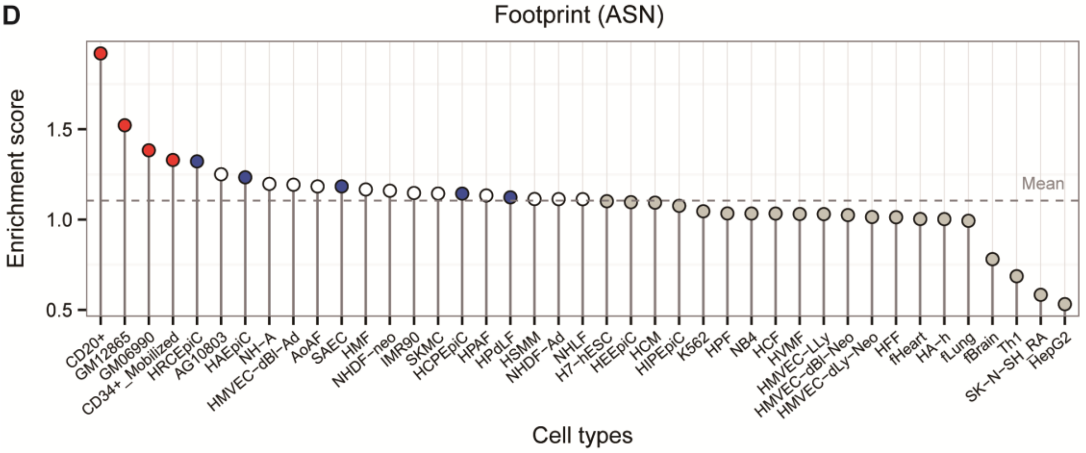

Footprint (ASN)
Enrichment score
1.5
1.0
0.5
Mean
Cell types
CD20+
GM12865
GM06990
CD34+_Mobilized
HRCEpiC
AG10803
HAEpiC
NH-A
HMVEC-dBl-Ad
AoAF
SAEC
HMF
NHDF-neo
IMR90
SKMC
HCPEpiC
HPAF
HPdLF
HSMM
NHDF-Ad
NHLF
H7-hESC
HEEpiC
HCM
HIPEpiC
K562
HPF
NB4
HCF
HVMF
HMVEC-LLy
HMVEC-dBl-Neo
HMVEC-dLy-Neo
HFF
fHeart
HA-h
fLung
fBrain
Th1
SK-N-SH_RA
HepG2

E

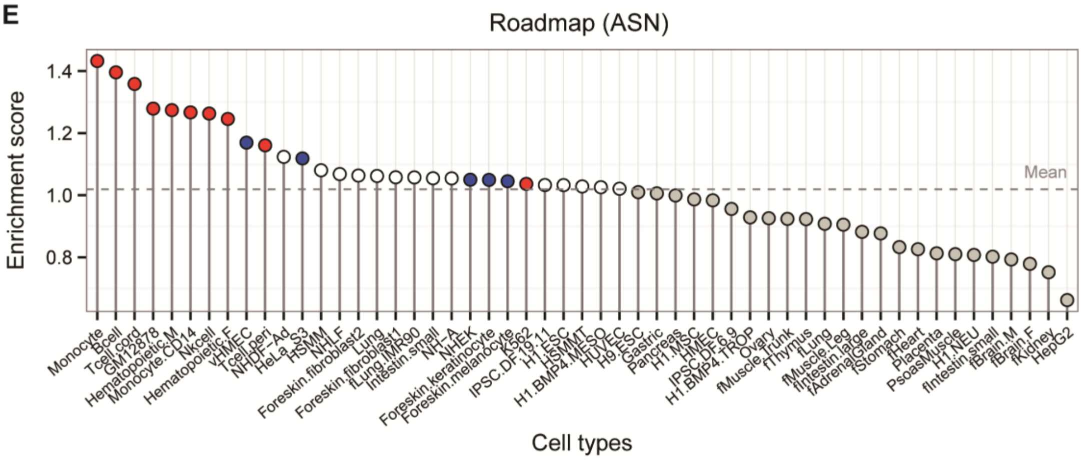

Roadmap (ASN)
Enrichment score
1.4
1.2
1.0
0.8
Mean
Cell types
Monocyte
Bcell
Tcell.cord
GM12878
Hematopoietic.M
Monocyte.CD14
Nkcell
Hematopoietic.F
vHMEC
Tcell.peri
NHDF-Ad
HeLa_S3
HSMM
NHLF
Foreskin.fibroblast2
Lung
Foreskin.fibroblast1
fLung.IMR90
Intestin.small
NH-A
NHEK
Foreskin.keratinocyte
Foreskin.melanocyte
K562
IPSC.DF.19.11
H1.ESC
HSMMT
H1.BMP4.MESO
HUVEC
H9.ESC
Gastric
Pancreas
H1.MSC
HMEC
IPSC.DF.6.9
H1.BMP4.TROP
Ovary
fMuscle.Trunk
fThymus
fLung
fMuscle.Leg
fIntestin.large
fAdrenalGland
fStomach
fHeart
Placenta
PsoasMuscle
H1.NEU
fIntestin.small
fBrain.M
fBrain.F
fKidney
HepG2

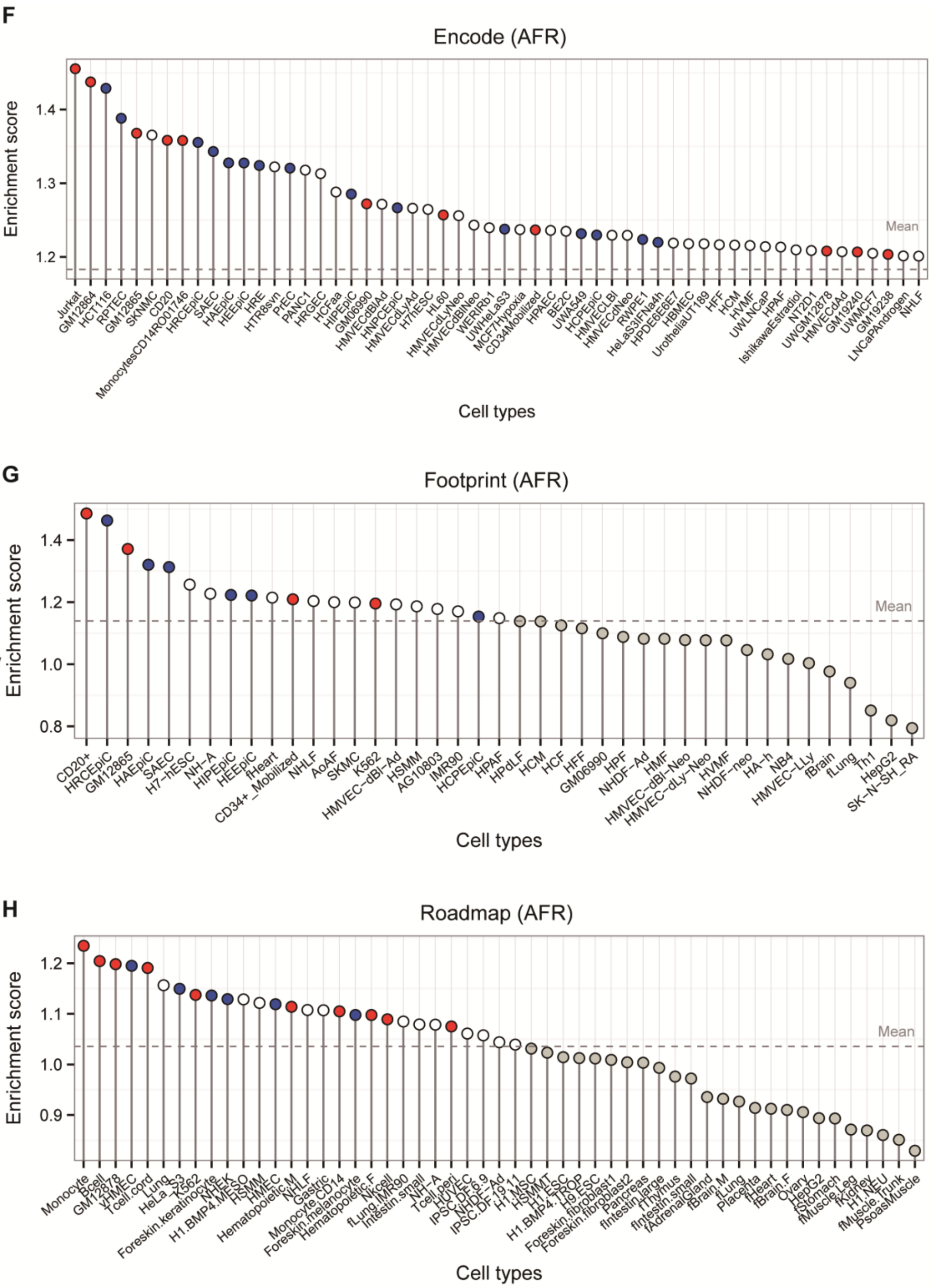
F
Encode (AFR)
Enrichment score
1.4
1.3
1.2
Mean
Jurkat
GM12864
HCT116
RPTEC
GM12865
SKNMC
MonocytesCD14RO01746
CD20
HRCEpiC
SAEC
HAEpiC
HEEpiC
HRE
HTR8svn
PrEC
PANC1
HRGEC
HCFaa
HIPEpiC
GM06990
HMVECdBlAd
HNPCEpiC
HMVECdLyAd
H7hESC
HL60
HMVECdLyNeo
HMVECdBlNeo
WERIRb1
UWHeLaS3
MCF7Hypoxia
CD34Mobilized
HPAEC
BE2C
UWA549
HCPEpiC
HMVECLBl
HMVECdNeo
RWPE1
HeLaS3IFNa4h
HPDE6E6E7
HBMEC
UrotheliaUT189
HFF
HCM
HVMF
UWLNCaP
HPAF
IshikawaEstradiol
NT2D1
UWGM12878
HMVECdAd
GM19240
UWMCF7
GM19238
LNCaPAndrogen
NHLF
Cell types
G
Footprint (AFR)
Enrichment score
1.4
1.2
1.0
0.8
Mean
CD20+
HRCEpiC
GM12865
HAEpiC
SAEC
H7-hESC
NH-A
HIPEpiC
HEEpiC
fHeart
CD34+_Mobilized
NHLF
AoAF
SKMC
K562
HMVEC-dBl-Ad
HSMM
AG10803
IMR90
HCPEpiC
HPAF
HPdLF
HCM
HCF
HFF
GM06990
HPF
NHDF-Ad
HMF
HMVEC-dBl-Neo
HMVEC-dLy-Neo
HVMF
NHDF-neo
HA-h
NB4
HMVEC-LLy
fBrain
fLung
Th1
HepG2
SK-N-SH_RA
Cell types
H
Roadmap (AFR)
Enrichment score
1.2
1.1
1.0
0.9
Mean
Monocyte
Bcell
GM12878
vHMEC
Tcell.cord
Lung
HeLa_S3
K562
Foreskin.keratinocyte
NHEK
H1.BMP4.MESO
HSMM
HMEC
Hematopoietic.M
NHLF
Gastric
Monocyte.CD14
Foreskin.melanocyte
Hematopoietic.F
Nkcell
fLung.IMR90
Intestin.small
NH-A
Tcell.peri
HUVEC
IPSC.DF.6.9
NHDF-Ad
IPSC.DF.19.11
H1.MSC
HSMMT
H1.ESC
H1.BMP4.TROP
H9.ESC
Foreskin.fibroblast1
Foreskin.fibroblast2
Pancreas
fIntestin.large
fThymus
fIntestin.small
fAdrenalGland
fBrain.M
fLung
Placenta
fHeart
fBrain.F
Ovary
HepG2
fStomach
fMuscle.Leg
fKidney
H1.NEU
fMuscle.Trunk
PsoasMuscle
Cell types

27

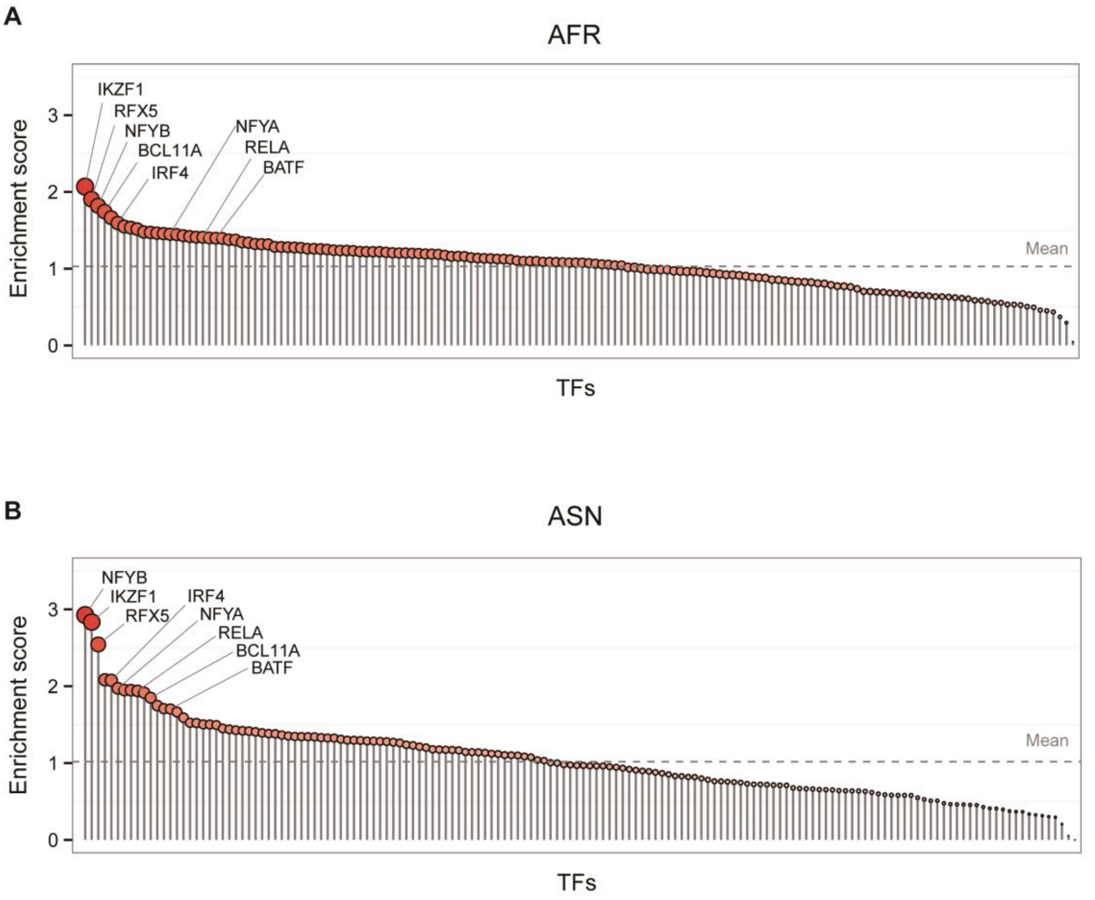

Figure S6. Enrichment of the balanced SNPs in TF binding regions. Cleveland's dot plots showing enrichment of the balanced SNPs in the binding regions of 161 TFs in the (A) ASN and (B) AFR panel. The same plot for the EUR panel is provided in Fig. 1C. The point size is proportional to the enrichment score. Only the top 50 TFs were displayed for the (C) EUR, (D) ASN, and (E) AFR population.

C

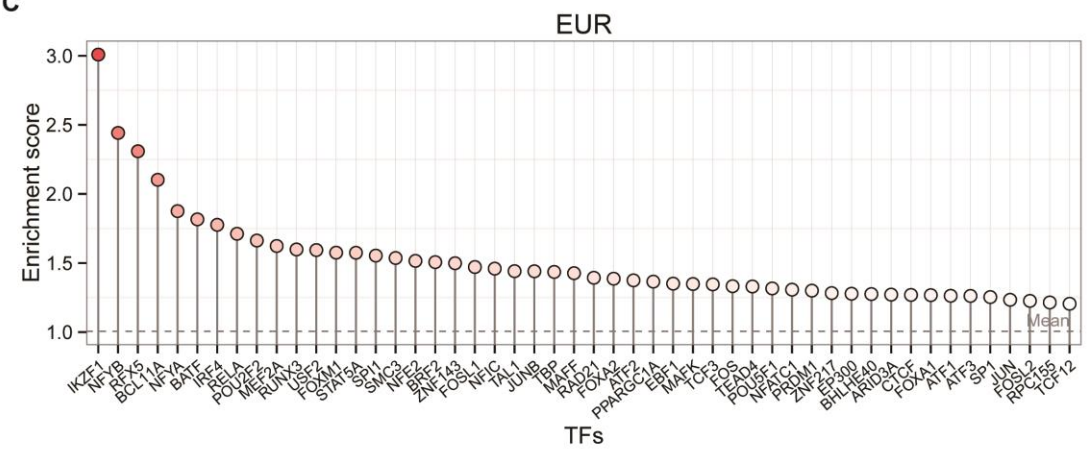
EUR
Enrichment score
3.0
2.5
2.0
1.5
1.0
Mean
IKZF1
NFYB
RFX5
BCL11A
NFYA
BATF
IRF4
RELA
POU2F2
MEF2A
RUNX3
USF2
FOXM1
STAT5A
SPI1
SMC3
NFE2
BRF2
ZNF143
FOSL1
NFIC
TAL1
JUNB
TBP
MAFF
RAD21
FOXA2
ATF2
PPARGC1A
EBF1
MAFK
TCF3
FOS
TEAD4
POU5F1
NFATC1
PRDM1
ZNF217
EP300
BHLHE40
ARID3A
CTCF
FOXA1
ATF1
ATF3
SP1
JUN
FOSL2
RPC155
TCF12
TFs

D

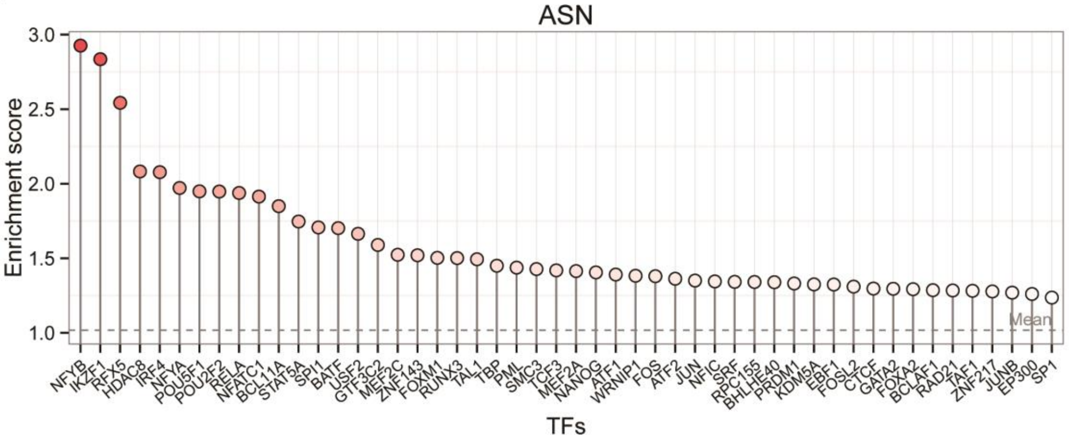
ASN
Enrichment score
3.0
2.5
2.0
1.5
1.0
Mean
NFYB
IKZF1
RFX5
HDAC8
IRF4
NFYA
POU5F1
POU2F2
RELA
NFATC1
BCL11A
STAT5A
SPI1
BATF
USF2
GTF3C2
MEF2C
ZNF143
FOXM1
RUNX3
TAL1
TBP
PML
SMC3
TCF3
MEF2A
NANOG
ATF1
WRNIP1
FOS
ATF2
JUN
NFIC
SRF
RPC155
BHLHE40
PRDM1
KDM5A
EBF1
FOSL2
CTCF
GATA2
FOXA2
BCLAF1
RAD21
TAF1
ZNF217
JUNB
EP300
SP1
TFs

E

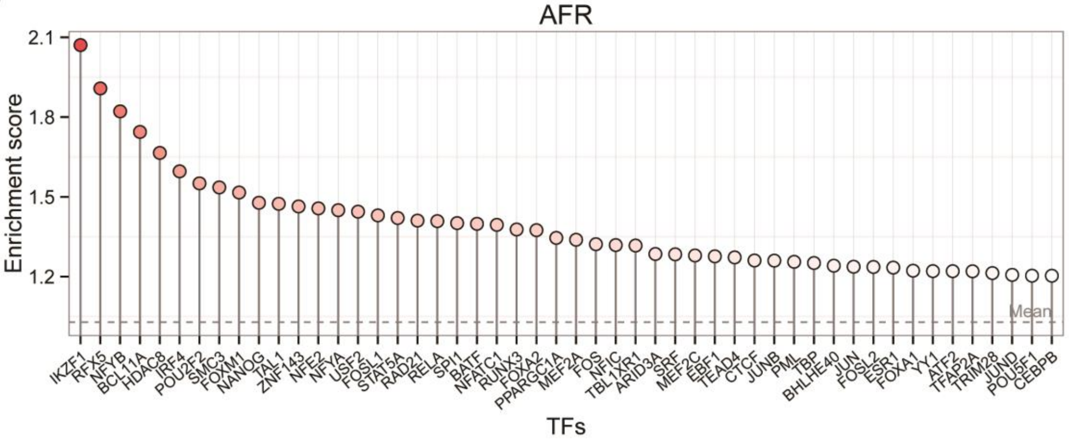
AFR
Enrichment score
2.1
1.8
1.5
1.2
Mean
IKZF1
RFX5
NFYB
BCL11A
HDAC8
IRF4
POU2F2
SMC3
FOXM1
NANOG
TAL1
ZNF143
NFE2
NFYA
USF2
FOSL1
STAT5A
RAD21
RELA
SPI1
BATF
NFATC1
RUNX3
FOXA2
PPARGC1A
MEF2A
FOS
NFIC
TBL1XR1
ARID3A
SRF
MEF2C
EBF1
TEAD4
CTCF
JUNB
PML
TBP
BHLHE40
JUN
FOSL2
ESR1
FOXA1
YY1
ATF2
TFAP2A
TRIM28
JUND
POU5F1
CEBPB
TFs

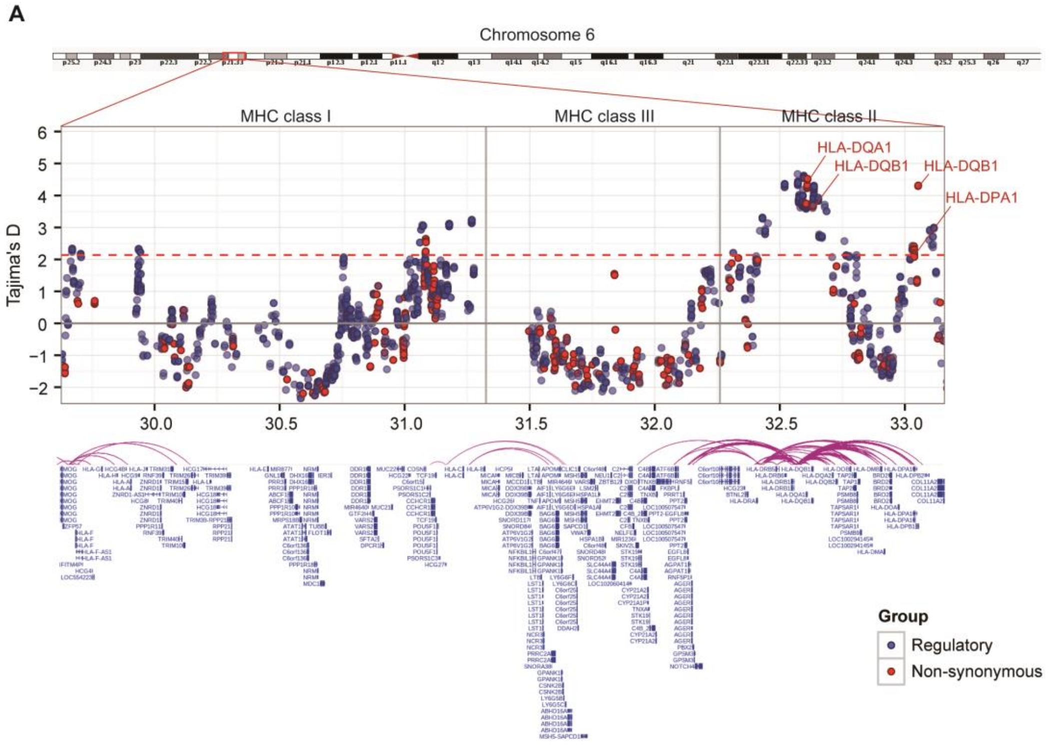

Figure S7. Chromosomal distribution of balancing selection signatures for MHC class II and SPRR/LCE genes. (A) Tajima's D signals at the MHC locus on chromosome 6 between the genetic markers MOG and COL11A2 (from 6p22.1 to 6p21.3 on hg19) for regulatory (blue) and non-synonymous SNPs (red). The horizontal dotted red line indicates the cutoff used for whole-genome detection of balanced SNPs (top 1% D). The promoter-enhancer chromatin interactions (Methods) were visualized by using the WashU Epigenome Browser (http://epigenomegateway.wustl.edu/browser/) in the bottom panel. (B) Tajima's D signals in the chromosome 1q21 region containing genes involved in epidermal differentiation for regulatory (blue) and non-synonymous SNPs (red). The chromatin interactions were displayed by using the WashU Epigenome Browser. (C) A zoom-in view of (B) into chr1:152350000-153250000.

B

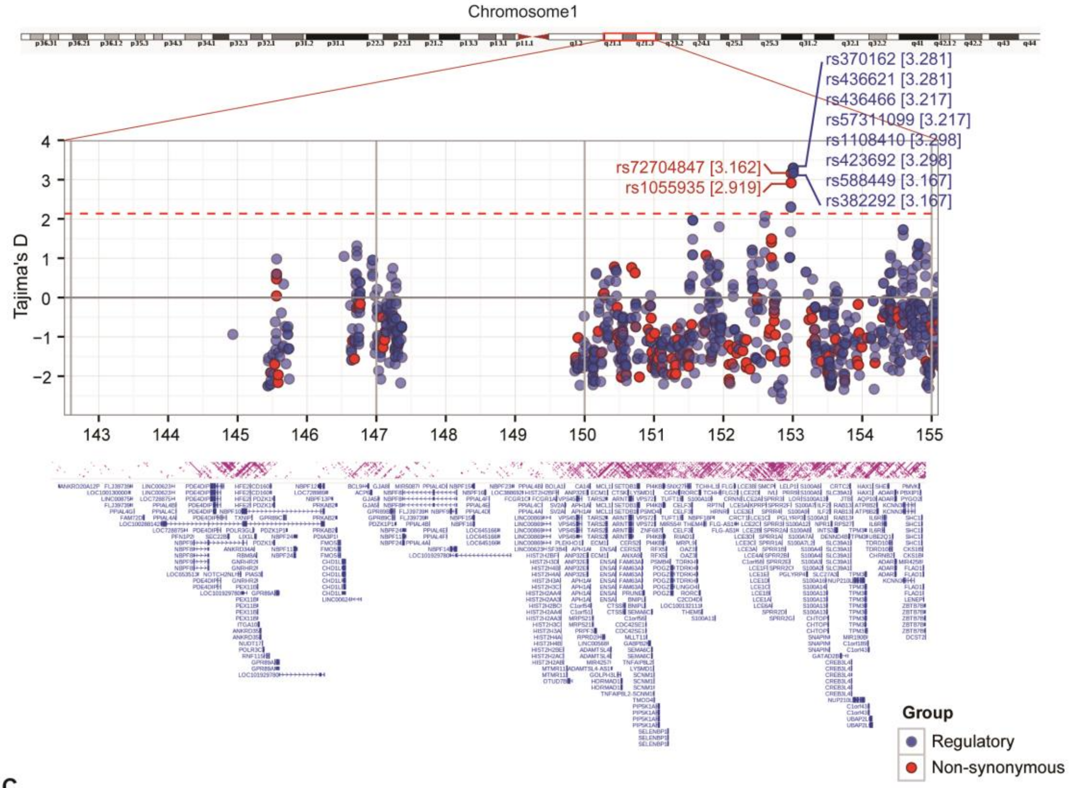
Chromosome1
rs370162 [3.281]
rs436621 [3.281]
rs436466 [3.217]
rs57311099 [3.217]
rs1108410 [3.298]
rs423692 [3.298]
rs588449 [3.167]
rs382292 [3.167]
rs72704847 [3.162]
rs1055935 [2.919]
Tajima's D
4
3
2
1
0
−1
−2
143
144
145
146
147
148
149
150
151
152
153
154
155
Group
Regulatory
Non-synonymous

C

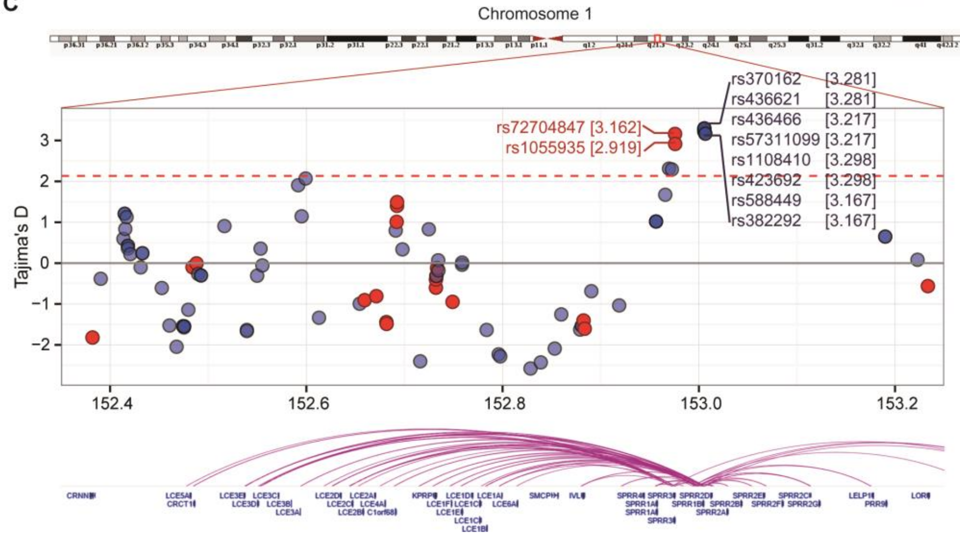
Chromosome 1
rs370162 [3.281]
rs436621 [3.281]
rs436466 [3.217]
rs57311099 [3.217]
rs1108410 [3.298]
rs423692 [3.298]
rs588449 [3.167]
rs382292 [3.167]
rs72704847 [3.162]
rs1055935 [2.919]
Tajima's D
3
2
1
0
−1
−2
152.4
152.6
152.8
153.0
153.2

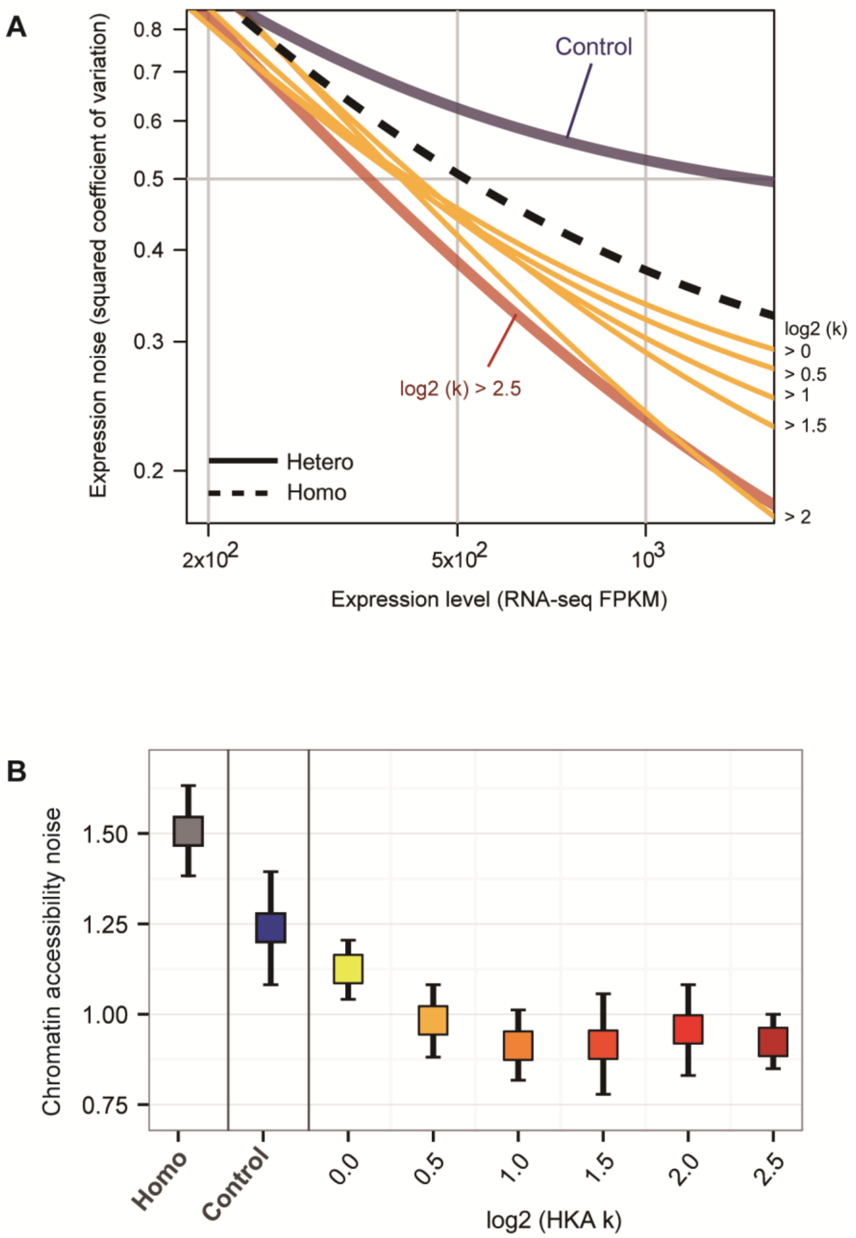

Figure S8. Noise in gene expression and chromatin accessibility as a function of HKA k. (A) Progressive reduction of the expression noise (CV^2) in proportion to the HKA k of the associated regulatory SNP. The curves show a fit to RNA sequencing data for 62 single cells with the solid and dashed lines representing heterozygous and homozygous loci in GM12878, respectively. The heterozygous curves are divided by the HKA k levels. (B) Cell-to-cell variability in chromatin accessibility for accessible regions categorized according to the HKA k levels in the same manner as in (A). Error bars represent one standard deviation of the variability obtained by bootstrapping cells (see Methods).

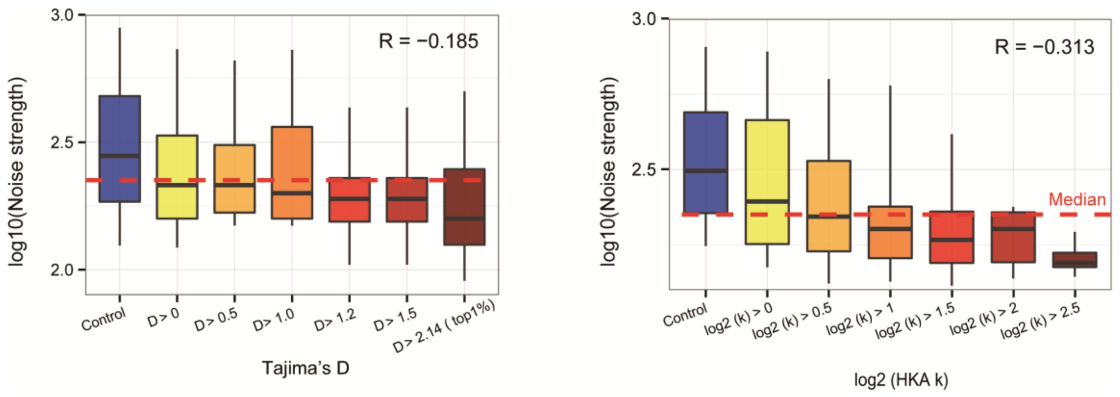

Figure S9. Negative correlation between the selection strength and gene expression noise. The noise strength of target gene expression as measured based on GM12878 single-cell RNA sequencing according to the selection strength at the corresponding regulatory SNPs as estimated by Tajima's D (left) or HKA k (right). Only heterozygous loci in GM12878 were used for the analyses.

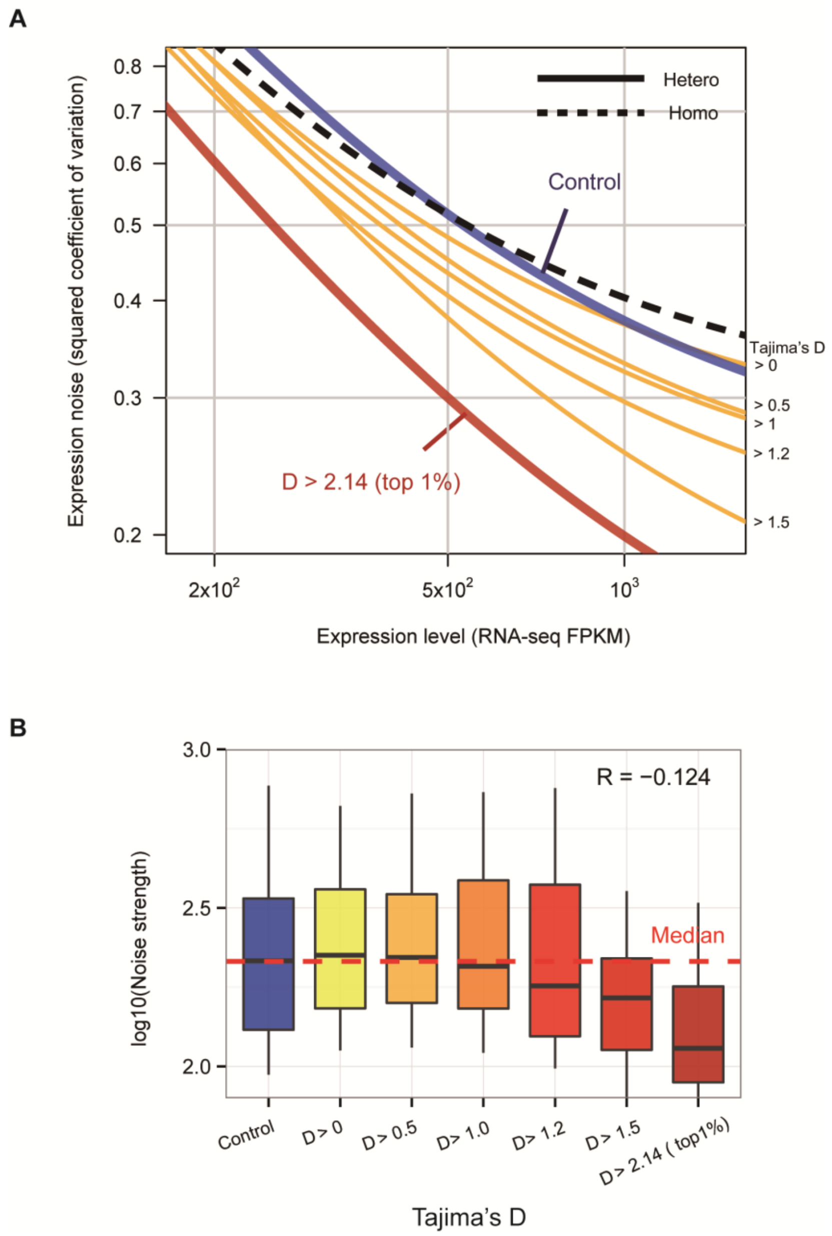

Figure S10. Lower expression noise of genes mapped to footprint SNPs by a different chromatin interaction dataset. (A) Progressive reduction of the expression noise (CV^2) in proportion to the HKA k of the associated regulatory SNP. The curves show a fit to GM12878 single-cell RNA sequencing with the solid and dashed lines representing heterozygous and homozygous loci in the GM12878 cells, respectively. The heterozygous curves are divided by Tajima's D. (B) The noise strength of target gene expression as measured based on GM12878 single-cell RNA sequencing according to the selection strength at the corresponding regulatory SNPs that are heterozygous in GM12878.

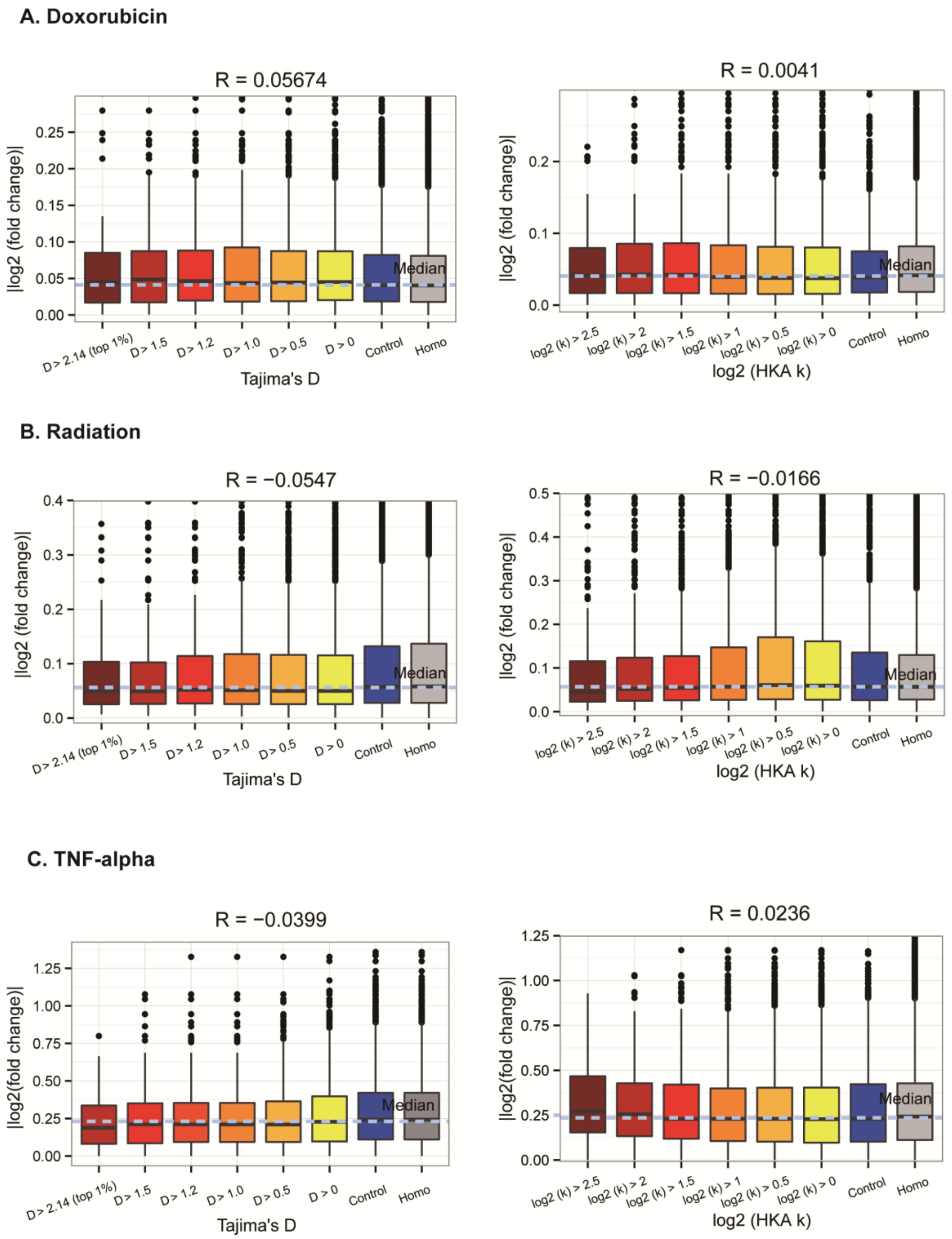

Figure S11. No association between the selection strengths at footprint SNPs and transcriptional responsiveness. (A-B) Transcriptional responsiveness of each gene was estimated as the change in gene expression level in response to the treatment of (A) doxorubicin or (B) ionizing radiation. The maximum change was obtained from multiple treatment experiments or time points. (C) Change in chromatin accessibility was calculated as the fold change of peak density in response to TNFα. (A-C) The grouping was based on Tajima's D (left) or HKA k (right) for the associated regulatory SNPs.
